# Proteomic insights into the physiology and metabolism of oleaginous yeasts and filamentous fungi

**DOI:** 10.3389/fmicb.2025.1637123

**Published:** 2025-09-05

**Authors:** Austin Gluth, Jesse B. Trejo, Jeffrey J. Czaijka, Shuang Deng, Wei-Jun Qian, Bin Yang, Tong Zhang

**Affiliations:** ^1^Biological Sciences Division, Pacific Northwest National Laboratory, Richland, WA, United States; ^2^Department of Biological Systems Engineering, Washington State University, Richland, WA, United States; ^3^Energy and Environment Directorate, Pacific Northwest National Laboratory, Richland, WA, United States; ^4^Agile BioFoundry, U.S. Department of Energy, Emeryville, CA, United States

**Keywords:** proteomics, oleaginous, yeast, fungi, bioproducts, oleochemicals, lipid production, stress response

## Abstract

Fungi are vital to the bioeconomy, serving as key producers of food, beverages, biofuels, and medicines, while also acting as essential resource recyclers in ecosystem management. For nearly a century, oleaginous yeast and filamentous fungi have been explored for their proficiency in oleochemicals production and carbon storage. Lipogenesis is one of the most well-studied fungal processes, with substantial progress having been made through reductionist biochemical approaches; however, the physiology and metabolism of fungal systems operating under different conditions arise from the functions of thousands of proteins, for which very little is known outside of model yeast. In this review, we discuss how proteomics provides a valuable analytical approach to contextualize lipogenesis within a complex biological system, where lipid accumulation is fundamentally governed by changes in proteins of multiple pathways. In the past two decades, proteomics has been applied to study stress response to nutrient limitations, metabolism of various carbon and nitrogen sources, the lipid droplet hub of carbon storage, protein post-translational modifications and signaling pathways, as well as oleochemical biosynthesis, thereby advancing our understanding of the oleaginous phenotype. Over 40 studies are reviewed herein to evaluate the impact, critically assess the utility, and propose future applications of proteomics. In the coming years, large systems-level proteomics studies will lay a foundation for marrying modeling and metabolic engineering strategies to optimize oleochemicals production in oleaginous fungi.

## 1 Introduction

**Figure 1:**
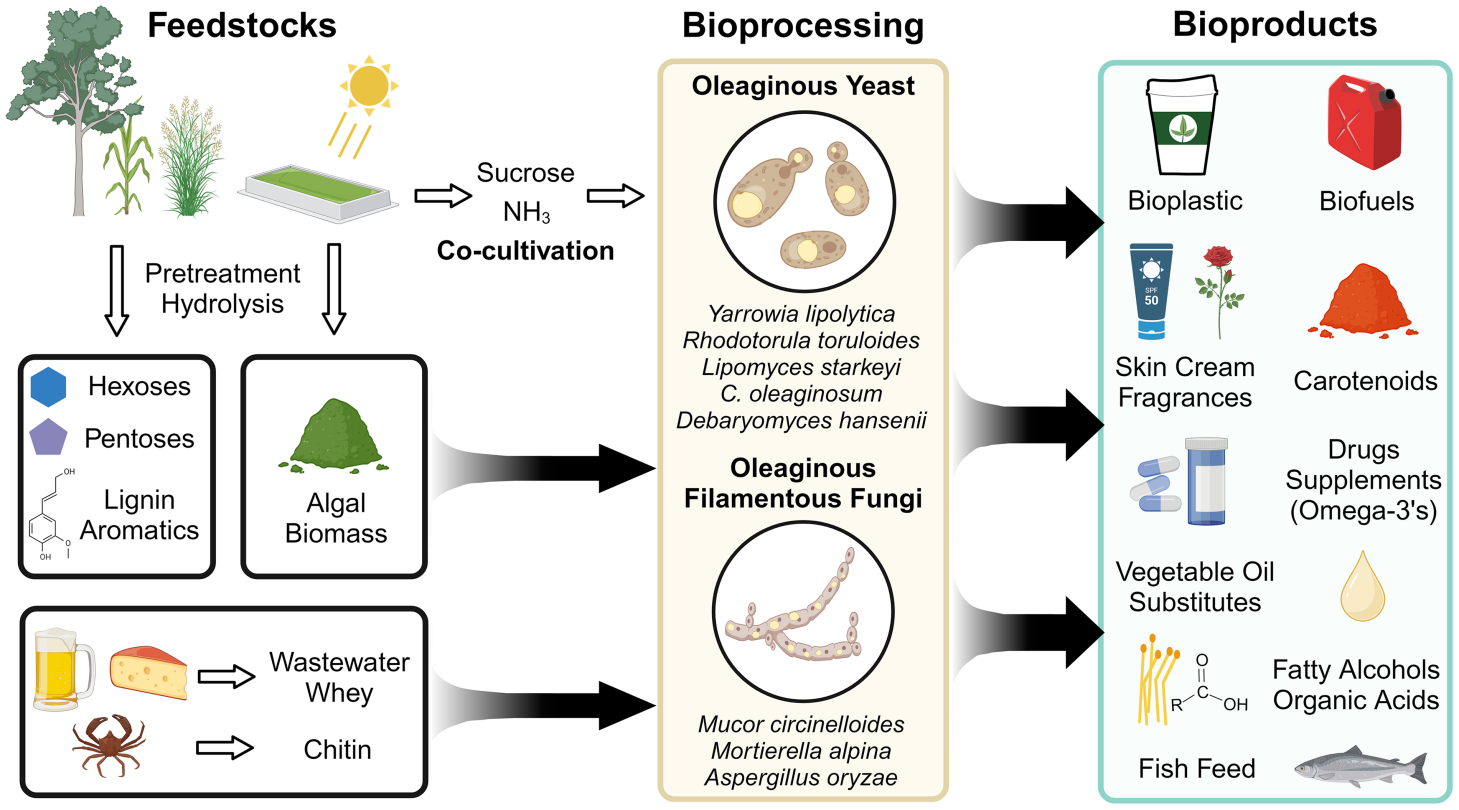
The potential of oleaginous fungi as part of a sustainable bioeconomy.

Ubiquitous in nature, fungi have evolved diverse metabolic capabilities for survival in a spectrum of niches. Certain fungi including single-celled yeast and multicellular filamentous fungi have developed a strategy to store surplus carbon as neutral lipids (triacylglyerides and sterols) in the face of competition or nutrient limitation (Mason-Jones et al., 2021). Strains capable of producing ≥ 20% of their cell dry weight as lipids are termed “oleaginous” and have been studied for three-quarters of a century (Lundin, 1950; Kessell, 1968; Gill et al., 1977; Botham and Ratledge, 1979; Higashiyama et al., 1999; Song et al., 2001). Oleaginous fungi have garnered sustained interest because of their potential as secure sources of oleochemicals (i.e., chemicals derived from natural oils) for biofuels, bioplastics, surfactants, and drug delivery vehicles (Figure 1) (Abeln and Chuck, 2021; Alhattab et al., 2024). With overfishing depleting marine ecosystems and accelerated deforestation due to land-intensive vegetable oil production, oleaginous fungi also offer cheaper and less land-intensive alternatives for fish feed, palm oil, and cocoa butter (Blomqvist et al., 2018; Tramontin et al., 2019; Abeln and Chuck, 2021; Brunel et al., 2022, 2024; Zhang X. Y. et al., 2022; Sigtryggsson et al., 2023).

To develop economically viable bioprocesses using oleaginous fungi, the value of the product, productivity at commercially-relevant scales (g/L/day product), and feedstock cost must be considered (Banerjee and Singh, 2024). Additional factors that should be considered include product yield (g product / g carbon source), land usage (tons product / hectare of land harvested for a carbon source), and energy and water inputs (Banerjee and Singh, 2024; Collett et al., 2014; Davis et al., 2022; Bhatt et al., 2022; Sartaj et al., 2023; Chopra et al., 2020). Several bioprocesses target low-cost feedstocks to provide carbon (and other nutrients) for bioproducts synthesis by oleaginous fungi (Figure 1). Emerging strategies include using algal biomass, plastic wastes, and even carbon/nitrogen-capturing co-cultures to supply resources (Younes et al., 2020; Zhou et al., 2023; Mihreteab et al., 2021; Yen et al., 2015; Cheirsilp et al., 2012; Pomraning et al., 2024). However, the single largest feedstock is lignocellulosic biomass derived from bioenergy crops and agricultural/forestry industry residues (Bioenergy Technologies Office, 2024). Lignocellulose is primarily composed of cellulose, hemicellulose, and lignin. Cellulose and hemicellulose are polysaccharides containing glucose, xylose, arabinose, and other monomers, while lignin is a complex heterogeneous polymer containing interlinked phenolics (Vanholme et al., 2010). Many oleaginous fungi natively utilize the major constituents of lignocellulose and are resistant to inhibitors from pretreatment steps that depolymerize lignocellulose (e.g., acetic acid, furan, furfural, and vanillin), making them poised for valorization strategies (Spagnuolo et al., 2019; Hassane et al., 2024).

Though hundreds of strains of oleaginous fungi from various phyla have been identified and studied for their lipid-producing capabilities (Ayadi et al., 2018; Abeln and Chuck, 2021; Zhang X. Y. et al., 2022), only a selection have sequenced genomes and even fewer have an accompanying set of genetic engineering tools. For yeasts, these include certain strains of the ascomycetes *Yarrowia lipolytica* (Abdel-Mawgoud et al., 2018), *Lipomyces starkeyi* (Zhang L. et al., 2022; Czajka et al., 2024), *Debaryomyces hansenii* (Yaguchi et al., 2017a; Strucko et al., 2021), and *Candida tropicalis* (Craft et al., 2003; Zhang et al., 2020) as well as the basidiomycetes *Rhodotorula toruloides* (Yu and Shi, 2023)*, Rhodotorula glutinis* (Pi et al., 2018), and *Cutaneotrichosporon oleaginosum* (Bracharz et al., 2017; Stellner et al., 2023; Shaigani et al., 2023). For filamentous fungi, the mucoromycetes *Mucor circinelloides* (Nagy et al., 2017; Fazili et al., 2022b) and *Mortierella alpina* (Hao et al., 2016; Sakamoto et al., 2017; Zhang et al., 2023) have been investigated for production of polyunsaturated fatty acids (PUFAs) (Zhang X. Y. et al., 2022). Nitrogen limitation is the most popular strategy to induce lipid accumulation, though phosphate, sulfate, or iron limitation can also be used (with combinatorial effects observed by limiting multiple nutrients) (Granger et al., 1993; Wu et al., 2010, 2011; Nambou et al., 2014; Dzurendova et al., 2021; Wierzchowska et al., 2021). The earliest systems-level analyses of oleaginous *Y. lipolytica* grown in nitrogen-rich vs. limited conditions were conducted using transcriptomics (Morin et al., 2011; Poorinmohammad and Kerkhoven, 2021). However, these studies showed no significant changes in the transcriptional profiles of fatty acid biosynthesis genes in response to nitrogen limitation. This suggests that lipogenesis may primarily be regulated at the post-transcriptional level, leading to alterations in protein profiles that ultimately drive differences in lipogenesis and lipid storage mechanisms between oleaginous and non-oleaginous fungi.

**Figure 2:**
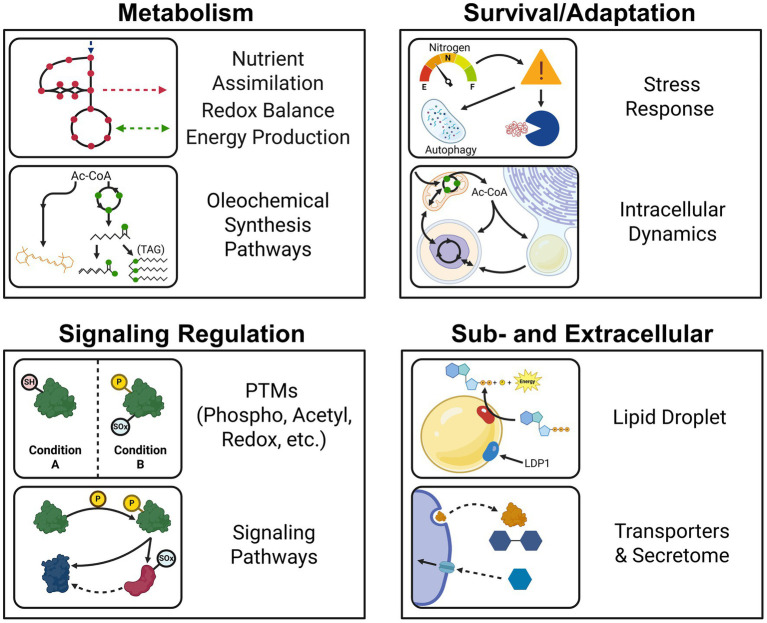
Summary of proteomics applications in studying the physiologies and metabolisms of oleaginous fungi.

Proteins are the major functional units in biological systems, and these functions ultimately give rise to phenotypes. Thus, studying proteins is key to understanding the unique characteristics of oleaginous fungi, and how they regulate lipid production in different environmental conditions. Biochemical tools for characterizing individual fungal proteins and genetically manipulating fungal strains have significantly advanced our understanding of enzyme function and central carbon metabolism (i.e., glycolysis/gluconeogenesis, pentose phosphate pathway or PPP, TCA cycle, and transhydrogenase cycle). However, these reductionist approaches fall short of capturing physiological snapshots of a biological system’s response to environmental conditions. In the past two decades, advances in mass spectrometry-based proteomics have enabled systems-level studies of proteins in oleaginous fungi. Unlike transcriptomics, which examines RNA expression, proteomics directly measures protein expression, offering a more precise view of cellular activity. As of writing, more than 40 publications have harnessed proteomics to investigate the physiologies of oleaginous fungi according to Pubmed and Web of Science™ (Dec, 2024). This body of work encompasses studies involving stress response to nutrient limitation, utilization of different carbon sources, the lipid droplet composition, secreted proteins (the secretome), protein modifications, comparisons among fungi, and integration with other omics approaches such as transcriptomics (transcriptional regulation), lipidomics (lipid profiles), and metabolomics (metabolites analysis) (Figure 2).

In this review, we provide a critical summary of proteomics studies in oleaginous fungi and highlight the role of proteomics in advancing our understanding of the oleaginous yeast phenotype. First, we provide an overview of the prevailing theory that explains in part how carbon flux is regulated and re-routed for lipid production in oleaginous fungi. Then, we summarize the overall proteomics workflow, and approaches that have been used to study oleaginous yeast and filamentous fungi. The core of our review focuses on proteomics insights into the unique physiology, metabolism, and stress responses mechanisms of these microorganisms (Figure 2). Lastly, we address challenges, offer recommendations, and identify unexplored opportunities to bolster proteomics applications for developing hyperlipogenic fungi.

## 2 Lipid accumulation in oleaginous fungi

**Figure 3:**
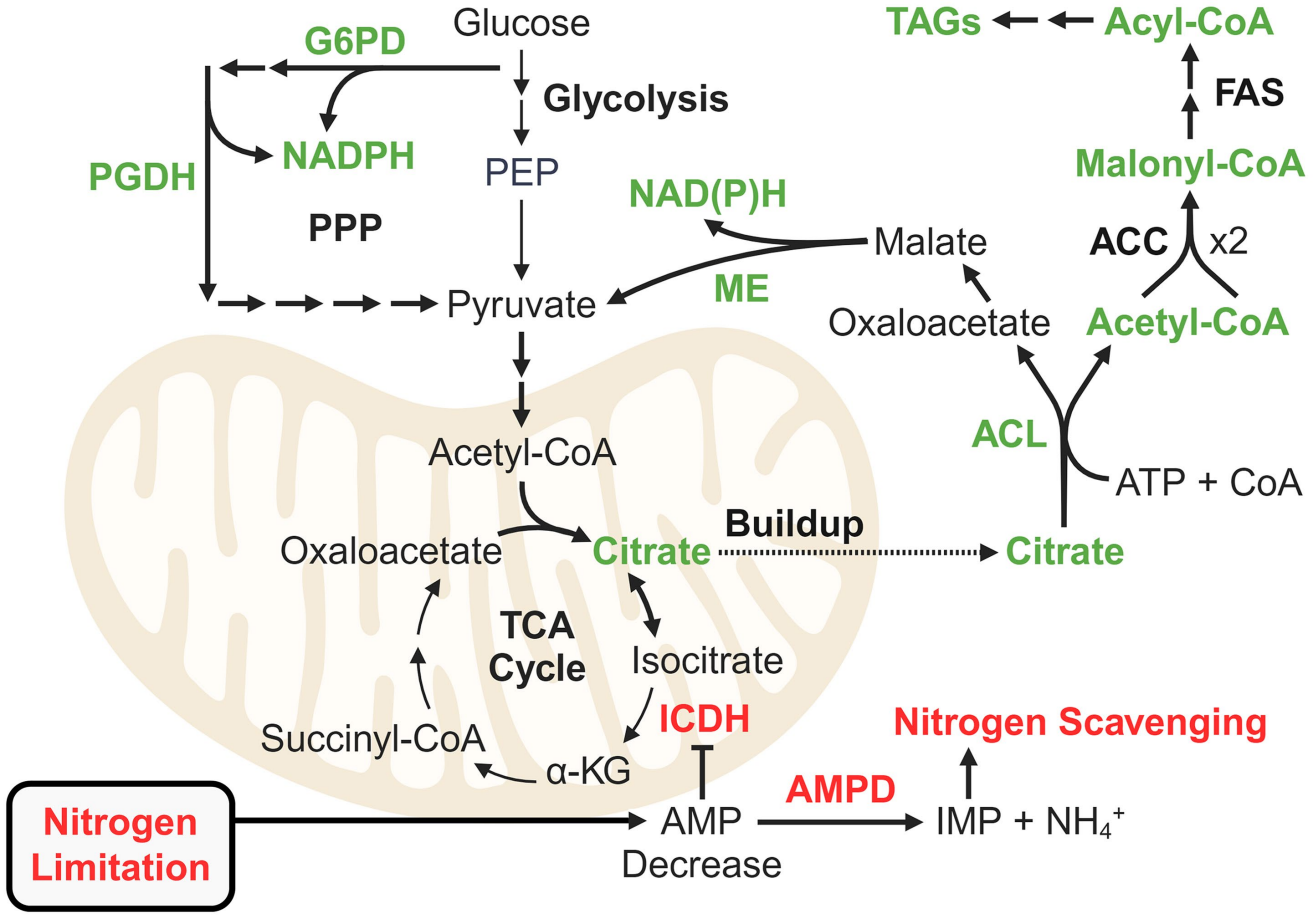
Theory of carbon re-routing for lipid accumulation in oleaginous fungi as a stress response to nutrient limitation. Green represents enzymes important for routing carbon flux to fatty acid biosynthesis. Red represents the regulatory mechanism linked to nitrogen recycling that leads to inhibition of isocitrate dehydrogenase and mitochondrial citrate buildup. Proteomics studies have elucidated additional pathways and reactions for supplying NADPH and acetyl-CoA for fatty acid biosynthesis. ACC: Acetyl-CoA carboxylase; ACL: ATP citrate lyase; AMPD: AMP deaminase; FAS: Fatty acid synthase; G6PD (GND1): Glucose-6-phosphate dehydrogenase; ICDH: Isocitrate dehydrogenase; ME: Malic enzyme; PGDH (ZWF1): 6-phosphogluconate dehydrogenase; PPP: Pentose phosphate pathway.

The mechanisms of lipid accumulation in oleaginous fungi and their environmental and metabolic triggers have been extensively reviewed (Beopoulos et al., 2011; Adrio, 2017; Spagnuolo et al., 2019; Abeln and Chuck, 2021; Zhang X. Y. et al., 2022). The current model for oleaginocity implicates triacylglycerides (TAGs) as sinks for carbon that would have otherwise been used for cell growth and division (Figure 3). When a key nutrient is limiting, flux through anabolic growth-promoting processes is generally downregulated, thereby severely perturbing cellular energy homeostasis and resulting in inhibition of key enzymes such as isocitrate dehydrogenase (ICDH) (Evans and Ratledge, 1985a,b). Nitrogen limitation leads to a decrease in cellular AMP levels, which is exacerbated by (though not entirely due to) the activity of the nitrogen scavenger and regulator AMP deaminase (Evans and Ratledge, 1985a,b). As an allosteric positive regulator of ICDH, a decrease in AMP leads to excretion of citrate from the mitochondria. ATP citrate lyase (ACL) then converts citrate into acetyl-CoA and oxaloacetate, fueling lipid biosynthesis (Ratledge and Wynn, 2002).

Fatty acid biosynthesis requires substantial NADPH, which, in oleaginous fungi like *Rhodosporidium toruloides* and *Mucor circinelloides*, is often supplied by malic enzyme (ME) (Ratledge, 2014). However, in species such as *Y. lipolytica* and *L. starkeyi*, ME does not produce NADPH, implicating alternative pathways (Tang et al., 2010; Zhang et al., 2013). The pentose phosphate pathway (PPP) is a well-studied source of NADPH, but other pathways such as the pyruvate-isocitrate-*α*-ketoglutarate cycle (involving NADP^+^-dependent ICDH) and the GABA shunt also play roles (Ratledge, 2014; Liu et al., 2019; Henriques et al., 2021). Moreover, it is clear that there are organism and condition-specific nuances regarding NADPH (and acetyl-CoA) generation. Such nuances have been revealed in recent proteomics studies, and insights beyond the hackneyed role of PPP (unless relevant to xylose utilization) will be explored for oleaginous yeast and filamentous fungi.

## 3 Proteomics approaches to study oleaginous fungi

A major goal of proteomics is to identify and understand proteins as a function of a biological system’s response to environmental stimuli (Li X. et al., 2021). Mass spectrometry (MS) is currently the premier platform for analyzing complex mixtures of proteins/peptides (Aebersold and Goodlett, 2001; Aebersold and Mann, 2003; Cox and Mann, 2011; Shuken, 2023). There are two general proteomics strategies: the bottom-up approach in which proteins are enzymatically digested into peptides prior to liquid chromatography (LC) MS analysis (LC–MS), and the top-down approach in which intact proteins are analyzed directly (Dupree et al., 2020). In both cases, peptides/proteins are separated by LC, followed by electrospray ionization (or matrix-assisted laser desorption/ionization, MALDI) to enter MS. Currently, the top-down approach has not been applied to oleaginous fungi and will not be discussed. For bottom-up proteomics studies of oleaginous fungi, data-dependent acquisition (DDA) is commonly used to collect MS spectra, which contain mass-to-charge (m/z) ratios at particular retention times (Li X. et al., 2021). A selection of peptides at a particular time are subjected to fragmentation (MS2 or tandem MS/MS) for confident identification of peptide sequences through proteome database searching. Additional details regarding proteomics methodologies are reviewed elsewhere (Jiang et al., 2024).

The bottom-up approach can be used to study the cellular proteome and the secretome containing extracellular proteins. To study intracellular proteins, fungi are first lysed via chemical (e.g., HCl), enzymatic (e.g., Zymolyase), and/or mechanical (e.g., bead-beating) means (Zhao et al., 2024). For filamentous fungi in particular, homogenization has often been employed for cell lysis due to their thick chitinous cell walls (Ling et al., 2015; Tang et al., 2016). Following lysis, proteins are solubilized and denatured by a variety of methods tailored to downstream sample processing steps (Awad and Brueck, 2020). One common approach involves using high concentrations of denaturing agents such as urea followed by dilution and digestion. For oleaginous fungi in particular, effort has been invested in solubilizing hydrophobic proteins and removing excess lipids using detergents (e.g., SDS and Triton X-100) and chloroform/methanol precipitation (Liu et al., 2009; Martinez-Moya et al., 2011; Kim et al., 2021). Compared to the intracellular proteome, proteins in the secretome are dilute and must be concentrated prior to sample processing using molecular weight cut-off filters or precipitation (Wei et al., 2013; Onésime et al., 2022). As with older cellular proteomics studies, some secretomics approaches employ SDS-PAGE for separation and in-gel digestion to acquire peptides for MS (Athenstaedt et al., 2006; Wei et al., 2013; Ciesielska et al., 2014).

**Figure 4:**
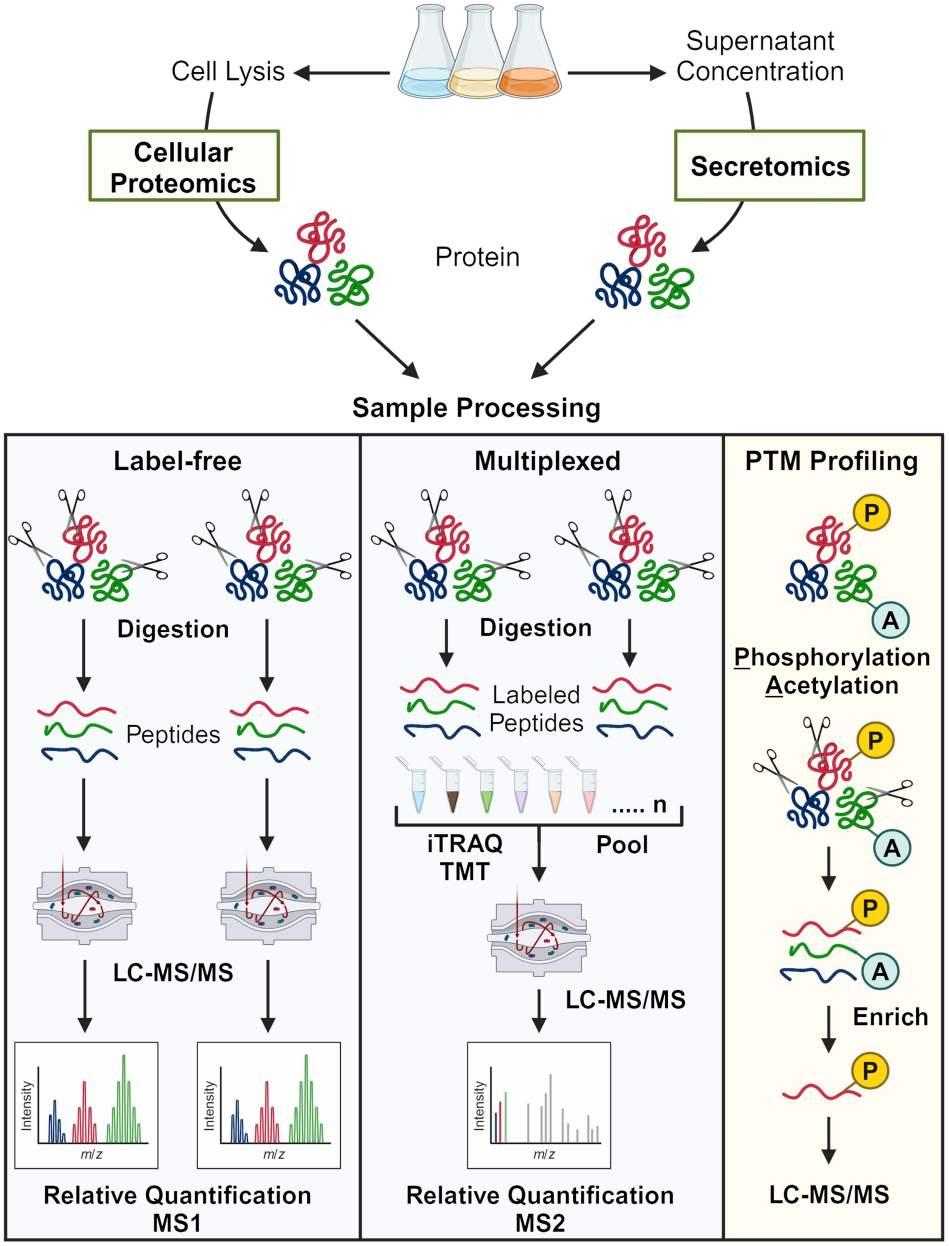
Overview of bottom-up proteomics approaches used to study oleaginous fungi. The two major quantification methods, label-free and peptide tagging (multiplexed), are shown. For PTM profiling, the selectivity of the enrichment method determines the major type of modified peptide that is enriched (generally one type). Note that many secretomics, lipid droplet proteomics, and early proteomics approaches employed SDS-PAGE and in-gel digestion prior to mass spectrometry analysis.

Proteomics strategies are further categorized according to quantification methodology (Figure 4). Label-free proteomics refers to workflows without peptide labeling. Relative quantification is achieved by comparing peptide spectrum matches (PSMs) from MS2 or extracted ion chromatography peak areas using MS1 intensity for a given protein across different conditions. Though relatively straightforward, label-free proteomics suffers from the missing data problem—peptides detected in one sample may not be detected in the other samples (Webb-Robertson et al., 2015). Multiplexed quantitative proteomics typically refers to workflows in which peptides are labeled with isobaric mass tags such as iTRAQ or TMT (Figure 4) (Pappireddi et al., 2019; Li J. et al., 2021). Labeling strategies such as stable isotope labeling with amino acids in cell culture (SILAC) (Ong et al., 2002) and stable-isotope dimethyl labeling (Hsu et al., 2003) also technically facilitate multiplexing, but few have applied these methods to oleaginous fungi (Tables 1, 2 and Supplementary Table 1).

Protein function is not solely attributed to abundance; rather, post-translational modifications (PTMs), protein–protein interactions, and subcellular localization also have important regulatory implications. PTMs such as phosphorylation and acetylation are labile and sub-stoichiometric, thus requiring additional sample processing considerations (e.g., phosphatase inhibitors) to avoid artifacts and enrich low-abundance PTMs. Enrichment strategies mainly include immunoprecipitation by PTM-specific antibodies (e.g., acetylation) and affinity capture techniques based on the chemical properties of PTMs (e.g., phosphorylation) (James Sanford and Bustamante Smolka, 2022). Though not summarized in Figure 4, proteins that localize to the lipid droplet are particularly interesting because of their structural and regulatory roles in TAG/sterol storage, lipid metabolism, and energy homeostasis (Athenstaedt, 2019). As with PTMs, additional steps are required to study protein localization and include, for instance, ultracentrifugation in the presence of high concentration salts or density gradients of sucrose or sorbitol (Athenstaedt et al., 2006; Zhu et al., 2015; Yu et al., 2017; Bhutada et al., 2018). All the methods summarized thus far have been applied to study oleaginous fungi; this proteomics anthology and the resulting physiological insights are reviewed hereafter.

## 4 Proteomics insights for oleaginous yeast

Proteomics enables us to decipher the expressed metabolism of oleaginous yeasts in response to environmental stimuli. Characterizing an organism’s expressed metabolism under different conditions reveals key enzymes and pathways that coordinate central metabolites for biosynthesis of lipids and other bioproducts, energy production, and redox balance. The proteomes of oleaginous yeast, especially *Y. lipolytica* and *R. toruloides,* have been studied more extensively than filamentous fungi and are reviewed hereafter (Table 1 and Supplementary Table 1, which includes all oleaginous yeast proteomics studies). Results from other biotechnologically-relevant oleaginous yeasts like *Lipomyces starkeyi* and *Cutaneotrichosporon oleaginosum* (formerly *Cryptococcus curvatus* and *Trichosporon oleaginosus*) are reviewed in the final portion of this section.

**Table 1:** Summary for select proteomics studies of oleaginous yeast.

Approach	Multi-omics	Stress	Organism	Carbon	Nitrogen	Experimental	Lipid titer/content	IDs	Significant findings summary	Ref.
Label-free cellular proteomics	G, T	Carbon	*C. oleaginosum*	Pretreated corn stover 1 g/L Aromatics 1 g/L Glucose	YE (NH_4_)_2_SO_4_	Carbon source conditions compared (mid-exponential phase cells); Batch flasks	0.24 g/L, 31.8% for alkaline-pretreated corn stover w/o (NH_4_)_2_SO_4_	-	First proteomics investigation of lignin-derived aromatics catabolism in oleaginous yeast. Enzymes of beta-ketoadipate pathway annotated.	Yaguchi (2020)
Label-free cellular proteomics	-	Nitrogen	*L. starkeyi*	70 g/L Glucose	YE (NH_4_)_2_SO_4_	Time course (8, 48, and 96 h); Batch bioreactor	0.5 g/L, 14%; 12.3 g/L, 28%; 30.0 g/L, 46%	> 250	Early proteomics results demonstrating upregulation of enzymes in PPP for regenerating NADPH and routing TCA derived carbon to fatty acid biosynthesis.	Liu et al. (2011)
Multiplexed cellular proteomics	L	-	*R. toruloides*	25 g/L Glucose	YNB (NH_4_)_2_SO_4_	Timecourse (24, 48, and 72 h); Nitrogen-rich vs. limited; Batch flasks	0.97 g/L, 10.1% High N; 2.27 g/L, 27.5% Low N	>23,000^PTM^ 2,804	First redox proteome of oleaginous yeast. Extensively detailed phosphorylation and protein oxidation patterns regulating lipogenesis and autophagy.	Gluth et al. (2025)
Multiplexed cellular proteomics	L, M, T	-	*R. toruloides*	10 g/L Glucose 10 g/L Xylose 10 g/L Arabinose 10 g/L Coumarate	YNB (NH_4_)_2_SO_4_ AA Mix	Timecourse (varying time points for each carbon source); Batch flasks	-	5,643	Multi-omics data used to improve the metabolic model for lignocellulose-derived carbon sources (xylose, arabinose, and p-coumarate).	Kim et al. (2021)
Label-free cellular proteomics	G, T	Nitrogen	*R. toruloides*	70 g/L Glucose	YE (NH_4_)_2_SO_4_	Timecourse (Seed†, 24 and 96 h); Batch bioreactor (Technical replicates?)	0.07 g/L, 22.8% High N; 0.16 g/L, 33.3% Low N	3,108	First systems-level construction of metabolism linked to oleaginous phenotype in this yeast. Found a new class of perilipin-like protein that likely protects lipid droplets.	Zhu et al. (2012)
Label-free lipid droplet proteomics	-	-	*Y. lipolytica*	20 g/L Glucose 5 g/L Oleic acid	YE Peptone YNB NH_4_Cl	Timecourse (3 and 24 h); YPD vs. minimal medium with oleic acid; Batch flasks	-	> 30^‡,§^	RAB GTPases involved in membrane trafficking, autophagy, etc. associated with LD. OIL1 protects LD from lipases.	Athenstaedt et al. (2006)
Multiplexed phospho	M	Nitrogen	*Y. lipolytica*	25 g/L Glucose	YNB (NH_4_)_2_SO_4_	9 h; Nitrogen rich vs. limited comparison; Batch flasks	-	1,219^PTM^ 4,926	Many phosphorylation sites of kinases not conserved in *S. cerevisiae*. RIM11 phosphorylation conserved and regulates glycogen synthesis.	Pomraning et al. (2016)

Full summary of proteomics studies in Supplementary Table 1. In the “Multi-omics” column, the letters correspond to the following: genomics (G), transcriptomics (T), metabolomics (M), and lipidomics (L). For filamentous fungi, PUFA lipid results are provided when specified. Note that the number of unique proteomics IDs is heavily influenced by sample processing, incorporation of offline fractionation to reduce sample complexity, and the MS searching algorithm (i.e., using MaxQuant with match-between-runs boosts IDs but many of them have missing values). † Seed culture medium (YPD) or medium for comparison differs substantially from the other media used in the experimentation. ‡ Exact number not reported but estimated from differential expression results or inferred from supplementary material. § SDS-PAGE gel bands. *PTM* Number of unique PTM sites (the number of protein IDs is provided below). ~ Exact number not reported but extracted from figure(s).

### 4.1 Yarrowia lipolytica

*Yarrowia lipolytica* is a model bio-oil producing ascomycete yeast, for which a number of strains have been isolated and engineered. Few of the wildtype strains are considered oleaginous with much of the genetic engineering conducted on strain W29 (a.k.a. CLIB89 and ATCC 20460) (Abeln and Chuck, 2021; Salvador López et al., 2022), for example, to confer non-native assimilation of monosaccharides (xylose, galactose, etc.) and disaccharides (fructose, cellobiose, etc.) (Guo et al., 2015; Lazar et al., 2015; Ledesma-Amaro et al., 2016; Hapeta et al., 2017). *Y. lipolytica* can utilize hydrophobic substrates (e.g., oleic acid) for growth, *ex novo* lipid synthesis, and bioproduct synthesis (e.g., citric acid), hence its species nomenclature (Madzak, 2021). It accomplishes this task by producing a complex extracellular bioemulsifier called liposan, which is comprised of proteins, exopolysaccharides, lipids, and other metabolites (Onésime et al., 2022). Proteomics investigations of *Y. lipolytica*, its derivatives, and other oleaginous strains have been conducted using various nutrient sources and stress conditions (Table 2 and Supplementary Table 1). These proteomic results played key roles in enhancing the understanding of the oleaginous phenotype and are detailed in the following subsections.

#### 4.1.1 The lipid droplet proteome

The earliest proteomics study of *Y. lipolytica* strain W29 was conducted in 2006 by Athenstaedt et al., who characterized its lipid droplet proteomes using either glucose or oleic acid as the carbon source (Athenstaedt et al., 2006). The authors isolated lipid particles and identified 21 proteins, involved in fatty acid activation, lipid and sterol metabolism, and transport processes (Athenstaedt et al., 2006). The low number of protein identifications was due to the study being performed at the early stage of proteomics. Interestingly, several Rab GTPases were only observed in the oleic acid condition; these enzymes are involved in trafficking membrane proteins and lipids. They may help facilitate lipid droplet interactions with other organelles (e.g., endosomes and lysosomes) and recruit proteins to a burgeoning lipid droplet (Liu et al., 2007; Bouchez et al., 2015). The authors also observed a protein without a homolog in *S. cerevisiae*—Bhutada et al. keenly noted this and found that deleting this protein (termed OIL1) eliminated the oleaginous phenotype (Bhutada et al., 2018). They concluded that OIL1 functioned similarly to perilipins found in higher eukaryotes and protects the lipid droplet from lipase activity.

#### 4.1.2 Molecular mechanisms of nitrogen limitation response

To investigate regulatory mechanisms governing nitrogen limitation-induced lipid accumulation, Pomraning et al. (2016) performed a multi-omics analysis of the metabolome, proteome, and phosphoproteome in *Y. lipolytica* under nitrogen-replete and limited conditions. Global proteomics showed an upregulation of proteins associated with proteolysis and downregulation of proteins in *β*-oxidation, amino acid metabolism, and translation after 9 h of nitrogen limitation. Given those results, they tested if inhibiting translation using cycloheximide would result in lipid accumulation and observed lipid droplet growth as a consequence, though to a lesser extent than nitrogen limitation (Pomraning et al., 2016). In agreement with earlier transcriptomics work (Morin et al., 2011), they did not observe differential expression of ATP-citrate lyase (ACL) and acetyl-CoA carboxylase (ACC) at the protein level, suggesting post-translational regulation of lipogenesis. Using phosphoproteomics, Pomraning et al. identified 599 proteins that were significantly enriched in tyrosine and serine/threonine kinase activity, with 80 and 53 phosphopeptides showing an increase or decrease, respectively, under nitrogen limitation (Pomraning et al., 2016). For example, they observed increased phosphorylation levels of ACL and ACC during nitrogen limitation. ACC is negatively regulated by AMP kinase (AMPK or SNF1 in yeast) by phosphorylation (Shi et al., 2014), which has been used to engineer constitutively active ACC (Qiao et al., 2015). Unless the observed sites are different from those regulated by SNF1, the increase in ACC phosphorylation seems counterintuitive—highlighting a challenge with using proteomics for discovery when so much relies on assumptions regarding homology of conserved sequences.

In. *S. cerevisiae*, response to the quantity and quality of nitrogen sources is regulated by nitrogen catabolite repression, which may be partially conserved in *Y. lipolytica* (Mazurie et al., 2005; Lavoie et al., 2009; Pomraning et al., 2017). When high-quality nitrogen like amino acids or ammonium is available, GATA-type transcription factors GLN3 and GAT1 are phosphorylated and remain bound to URE2 in the cytosol. Under poor nitrogen conditions, they localize to the nucleus and activate nitrogen utilization genes. Pomraning et al. did not report changes in phosphorylation for GLN3, GAT1, GCN4, TOR1, or GATA-type transcriptional repressors GZF3 and DAL80, which may be due to phosphoproteomic coverage limitations, the low abundance of phosphorylated proteins, or the selected time points for analysis (Pomraning et al., 2016). However, they did observe changes in protein abundances for predicted GATA family transcription factors (e.g., upregulation of GLN3 during nitrogen limitation). They also keenly demonstrated how proteomics can be used to identify DNA motifs that associate transcription factors with up- and downregulated proteins. Genes with a G[AC]TAAGC and [GA]TGAGTCA motifs were enriched for amino acid biosynthesis. The latter may be bound by Gcn4p, which activates expression of genes in response to amino acid starvation (Pomraning et al., 2016). Interestingly, genes with the [GA]TGAGTCA motif tended to be downregulated, suggesting the importance of autophagy for scavenging amino acids and nitrogen and/or regulatory responses to ammonium starvation that are unique to *Y. lipolytica*.

#### 4.1.3 Growth rate and lipid accumulation

Disentangling growth-dependent proteome changes during nutrient-limitation from those consequential to lipid accumulation remains a challenge. To explore the relationship between growth rate and lipid accumulation, Poorinmohammad et al. (2022) used chemostat *Y. lipolytica* cultures at different dilution rates and performed comparative proteomic analysis. They found that lower growth rates often lead to higher lipid yields and observed upregulation of fatty acid synthase (FAS), ACL, and ACC in low growth rate vs. high growth rate conditions. Using a linear modeling approach and comparisons to a non-lipid accumulating strain, they were able to tease out some enzymes that regulate lipid accumulation independent to some extent of growth rate. Outside of PPP, which had been detailed before proteomics studies (Wasylenko et al., 2015), they identified the role of NADPH-producing enzymes formate dehydrogenase (FDH) and isocitrate dehydrogenase (ICDH) in driving lipid biosynthesis. Additionally, downregulation of ER-plasma membrane tethering proteins and upregulation of ER stress proteins (specific targets not provided) suggested the involvement of unfolded protein response (UPR) activation in lipid accumulation. Upregulation of chaperone-mediated autophagy proteins highlighted a possible connection between ER stress, UPR, and selective autophagy in regulating lipid metabolism. Interestingly, they proposed an additional link to oxidative stress according to superoxide dismutase upregulation that may be explained by mitophagy during nitrogen limitation (Venditti and Di Meo, 2020). Lastly, Poorinmohammad et al. observed downregulation of high osmolarity glycerol response 1 (HOG1), a mitogen-activated protein kinase known for its role in osmotic stress, which likely plays a role in lipid homeostasis (Herrero-de-Dios et al., 2020; Poorinmohammad et al., 2022). They knocked out HOG1 which resulted in a 20% increase in lipid production, demonstrating the power of proteomics analysis when paired with chemostat growth studies and metabolic engineering (Poorinmohammad et al., 2022).

#### 4.1.4 Impacts of low-cost carbon sources: plastics and lignocellulosic biomass

Hydrophobic substrate utilization by *Y. lipolytica* has translated to growing research interest in hybrid processes to upcycle plastic wastes, few of which focus on testing yeast in general (Gluth et al., 2022). Walker et al. (2023) studied proteome changes resulting from cultivation on hydrocarbon-rich oil (C11–C28 alkanes and alkenes) generated from catalytic decomposition of polyethylene. To grow on this toxic substrate, the yeast allocated a substantial portion of its proteome towards expression of alkane-degrading enzymes including cytochrome P450 oxidases, alcohol/aldehyde dehydrogenases, alcohol oxidases, fatty acid ligases, and *β*-oxidation enzymes. They also noted significant upregulation of an oxysterol-binding protein that may play a role in intracellular hydrocarbon transport (Fukuda, 2023).

In another study, lignocellulosic substrate from hydrolysis of switchgrass was used to compare the proteomes of *Y. lipolytica* strain W29 to the isolate strain YB420, which unlike the former is capable of growth and lipid production using xylose (Walker et al., 2021). Strain W29 encodes the genes for xylose utilization (xylose reductase, xylitol dehydrogenase, and xylulokinase) (Rodriguez et al., 2016); however, they appear to be “silent,” whereas YB420 upregulated xylitol dehydrogenase, xylulokinase, and PPP enzymes providing NADPH for further xylose assimilation and lipid biosynthesis. As a potential result of redox imbalances, W29 secreted xylitol and degraded lipids as a carbon and energy source in stationary phase.

#### 4.1.5 Thiamine deficiency

Thiamine pyrophosphate (TPP, the active form of vitamin B1) is a coenzyme required for pyruvate dehydrogenase and *α*-ketoglutarate dehydrogenase activity. *Y. lipolytica* cannot natively synthesize thiamine and its absence leads to a downregulation of proteins in lipid biosynthesis and energy metabolism (particularly ATP synthase) (Walker et al., 2020). Using genomics, the authors identified the missing thiamine biosynthesis protein 13 (THI13) gene and engineered a strain capable of *de novo* thiamine biosynthesis, which yielded a significant although moderate increase in lipogenesis (~4% vs. ~1% lipid accumulation for the parental strain) in the absence of supplemented thiamine during nitrogen limitation. It follows that all studies using *Y. lipolytica* listed in Supplementary Table 1 include a source of vitamins—a cost that may not be required for other oleaginous yeast (Nutrition Reviews, 1958).

### 4.2 Rhodotorula toruloides

*Rhodotorula toruloides* (formerly *Rhodosporidium toruloides*) is a model oleaginous basidiomycete yeast with an expansive catalog of studies probing its lipid-accumulating, carotenoid-producing phenotype. Popular oleaginous strains for this yeast include NP11 (haploid derived ultimately from CGMCC 2.1389) (Li et al., 2007; Zhu et al., 2012) and IFO0880 (now NBRC 0880) (Coradetti et al., 2018), both of which naturally utilize xylose for growth and lipid accumulation compared to prominent oleaginous *Y. lipolytica* strains.

#### 4.2.1 Setting the stage for multi-omics investigations of oleaginicity

In an early seminal multi-omics study, Zhu et al. sequenced the genome of *R. toruloides* and performed comparative transcriptomic and proteomic analyses of cells cultured under nitrogen-limited conditions (Zhu et al., 2012). They detailed lipid metabolism (including a novel FAS) and repression of TOR1, a negative regulator of autophagy, in *R. toruloides* under nitrogen-limiting conditions. Consequently, they observed suppression of protein biosynthesis machinery alongside an activation of autophagy-related proteins such as vacuolar proteases and ATPases. Lipid synthesis-related proteins, including key enzymes in fatty acid biosynthesis (ACL1, ACC1, FAS1/2), were elevated when comparing YPD seed cultures to those grown in minimal medium with limited nitrogen. Upon close inspection of their results, abundance changes between the 24 h and 96 h time points in nitrogen-limited minimal medium were in fact minimal (< 2 fold) or insignificant for many enzymes involved in fatty acid synthesis. There were several enzymes in other pathways (e.g., the PPP with GND1 downregulated and ZWF1 upregulated) that were, nevertheless, differentially expressed.

Importantly, their work shows how proteomics provides information about protein expression in organelles, and how said changes in expression may affect their corresponding processes. Zhu et al. noted that a perilipin-like protein (LDP1) was highly expressed during lipid production, suggesting its involvement in lipid droplet stability and formation (Zhu et al., 2012). In a follow-up study, the authors characterized this yeast’s lipid droplet proteome confirming the localization of LDP1, which was indeed upregulated during nitrogen and, separately, phosphate limitation (Zhu et al., 2015). They also observed several metabolic enzymes including GND1 and glyceraldehyde 3-phosphate dehydrogenase that were localized to the lipid droplet yet not directly involved in lipid metabolism. Additionally, the study found an oxylipin-producing enzyme PpoA, which is involved in hormone-like signaling that may be linked to lipid homeostasis and signaling.

#### 4.2.2 Lipid accumulation induced by phosphate limitation

**Figure 5:**
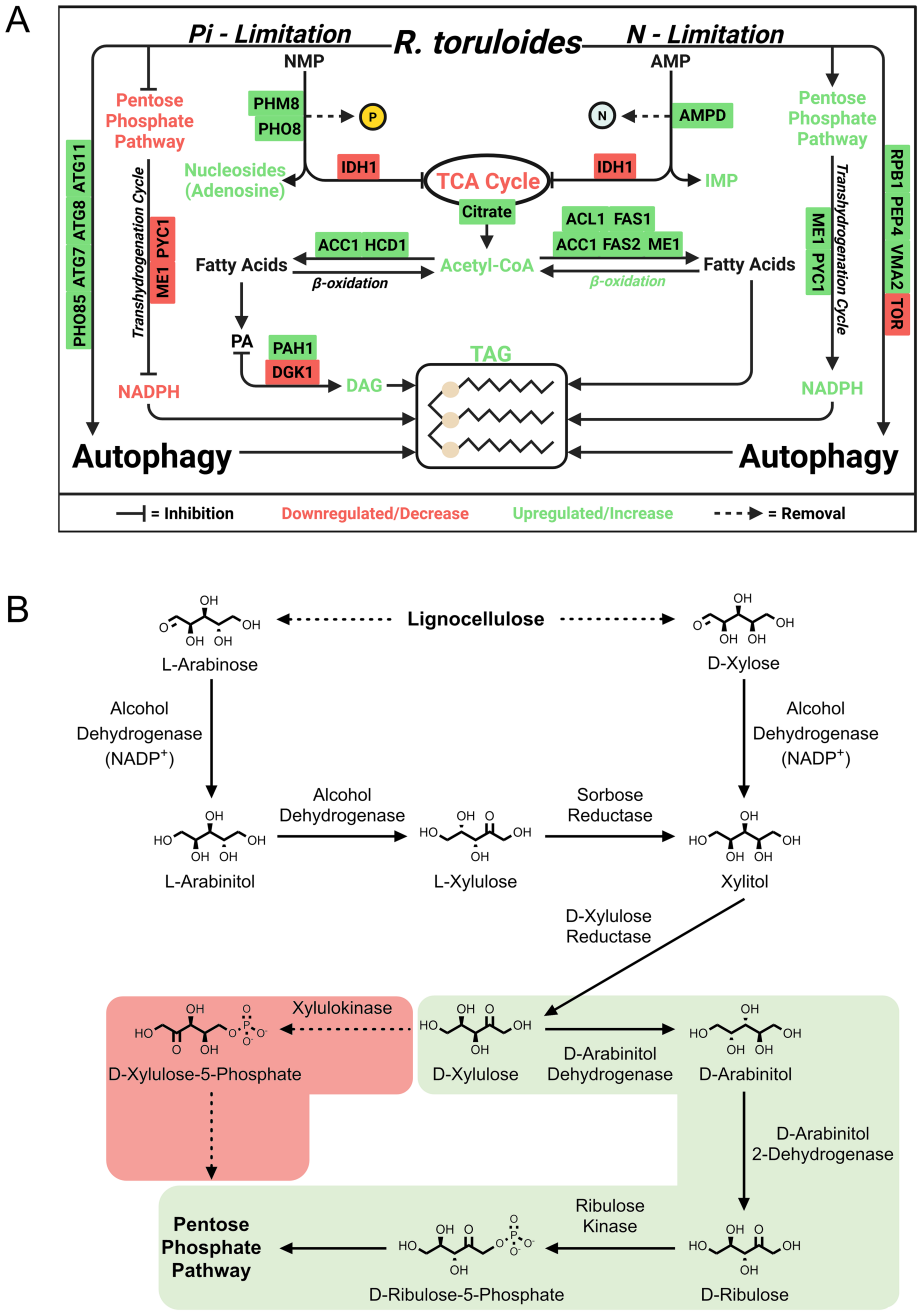
Examples of discoveries in *Rhodotorula toruloides* using proteomics. **(A)** Phosphate-limited regulation of lipid accumulation and stress response according to Wang et al. (2018, 2023a). **(B)** Xylose and arabinose metabolism as annotated by Kim et al. (2021). The light green shading indicates the correct pathway containing the intermediate D-arabinitol. ACC1, Acetyl-CoA carboxylase 1; ACL1, ATP citrate lyase 1; AGK1, Acylglycerol kinase 1; AMPD, AMP deaminase; ATG7, Autophagy-related protein 7; ATG8, Autophagy-related protein 8; ATG11, Autophagy-related protein 11; FAS1, Fatty acid synthase subunit beta; FAS2, Fatty acid synthase subunit alpha; HCD1, Hydroxyacyl-CoA dehydrogenase 1; ME1, Malic enzyme 1; PAH1, Phosphatidate phosphatase 1; PEP4, Proteinase A (vacuolar aspartyl protease); PHM8, Pyrimidine 5′-nucleotidase; PHO8, Vacuolar alkaline phosphatase; PHO85, Cyclin-dependent kinase; PYC1, Pyruvate carboxylase 1; RPB1, RNA polymerase II largest subunit; TOR, Target of rapamycin kinase; VMA2, Vacuolar H^+^-ATPase subunit B.

Some waste streams (e.g., those from food processing, distilleries, and anaerobic digestors) are rich in nitrogenous compounds, necessitating other strategies to induce lipid accumulation. This justified Wang et al.’s novel proteomics investigations of phosphate (inorganic phosphorus source, Pi) limitation (Wang et al., 2018, 2023a). In their first study (Wang et al., 2018), they found that Pi limitation activates the PHO pathway (phosphate homeostasis signaling), which triggers upregulation of Pi transporters, ribosome degradation, and RNA recycling to reclaim phosphate. In addition to key proteins involved in fatty acid biosynthesis, such as ACC1, upregulation of phosphatidate phosphatase and downregulation of DAG kinase routed carbon to lipid accumulation while limiting phospholipid production. Degradation of nucleotides appears to further connect phosphate limitation to lipid accumulation and was demonstrated by upregulation of phosphatases. Pi limitation leads to reduced AMP levels, shifting TCA cycle flux toward citrate accumulation (Figure 5A).

In a follow-up study (Wang et al., 2023a), the same group addressed the impacts of Pi limitation on the phosphoproteome, raising important regulatory considerations involving autophagy—rather than the PHO pathway directly. Phosphoregulation of autophagy-related proteins (ATG) is controlled by upstream regulators including TORC1, a nutrient-sensing negative regulator of autophagy and positive regulator of ribosome biogenesis, and SNF1, a positive regulator of autophagy. Because ATG9, an important regulator of autophagosome formation, was upregulated in protein abundance, the authors used RNA interference to silence its expression, which led to a ~ 50% decrease in lipid accumulation. Unfortunately, many of these proteins including TOR1 and ATG9 were not observed in either the global proteome (protein abundances) or phosphoproteome (PTM abundances) results, limiting conclusions that can be drawn about significant changes in protein phosphorylation that are independent of changes in protein abundances.

#### 4.2.3 Metabolic network analysis for lignocellulose-relevant carbon sources

To explore lignocellulosic carbon utilization, which includes appreciable quantities of glucose, xylose, and *p*-coumaric acid, Kim et al. reconstructed the metabolic network of *R. toruloides* using transcriptomics, proteomics, and metabolomics data (Kim et al., 2021). Importantly, they employed RB-TDNA sequencing to build a library of mutants to test fitness on these carbon sources. Consistent with a previous report that D-arabinitol was among the main fermentation byproducts from xylose in *R. toruloides* (Jagtap and Rao, 2018), their findings reveal that *R. toruloides* employs an alternative xylose utilization pathway in which D-xylulose is converted to D-arabinitol by a reductase and/or arabinitol dehydrogenase and subsequently converted to D-ribulose by a D-arabinitol 2-dehydrogenase (Figure 5B). D-ribulose is converted to D-ribulose-5-phosphate by D-ribulose kinase, which had significant fitness defects in the tested pentose sugar and alcohol media conditions.

A recent study by Alīna et al. used genome-scale metabolic modeling to explore metabolic trade-offs in *R. toruloides* related to lipid production using different carbon sources (glucose, xylose, and acetate) (Reķēna et al., 2023). They identified a key metabolic route for acetyl-CoA generation via ACL and the phosphoketolase pathway, which produces acetyl-phosphate and glyceraldehyde-3-phosphate. This pathway provides an engineering opportunity for carbon-efficient production of acetyl-CoA by bypassing CO_2_ generation from the pyruvate dehydrogenase step of glycolysis/TCA cycle (Hellgren et al., 2020).

#### 4.2.4 Xylose utilization and oxidative stress

Xylose utilization has been studied quite often in *R. toruloides* using proteomics. Tiukova et al. (2019) compared glucose vs. xylose-grown cells and observed upregulation of enzymes for xylose assimilation, such as xylitol dehydrogenase and several NADPH-dependent xylose reductases, as expected (Figure 5B). They also highlighted proteins involved in glutathione metabolism, oxidative stress response, and autophagy that were upregulated during nitrogen limitation in both conditions but also for the specific glucose vs. xylose comparison. The study revealed that peroxisomal *β*-oxidation was more pronounced in cells grown on xylose. Though not explicitly conjectured in their work, it is possible that H_2_O_2_ levels as a result of upregulated β-oxidation and competition between antioxidants and xylose reductase for NADPH partially explain those results.

Pinheiro et al. (2020) further investigated the role of oxidative stress on *R. toruloides*’ xylose metabolism and its role in enhancing lipid and carotenoid production. Oxidative stress, induced by H_2_O_2_ and indicated by a resulting upregulation of catalase, led to a marked increase in lipid content due in part to downregulation of fatty acid β-oxidation. They also reported that D-arabinitol was among the main fermentation byproducts, which agrees with an earlier study and raises questions regarding redox cofactor homeostasis because some sugar alcohols may be secreted to regenerate NAD(P)^+^ (Jagtap and Rao, 2018). Additionally, autophagy pathways and reduced amino acid biosynthesis under nitrogen depletion further enhance lipid accumulation, showing a tight coupling between oxidative stress responses and metabolic shifts during xylose fermentation.

Xylose is the primary constituent from dilute acid hydrolysis of lignocelluse. This and other pretreatment methods release additional compounds like vanillin, an aldehyde constituent of lignin, that are inhibitory and implicated in oxidative stress response in fungi (Kim et al., 2014; Nguyen et al., 2014). Qi et al. (2014) screened mutant strains with enhanced tolerance to sugarcane bagasse hydrolysate and, in a following study, used proteomics to compare the wild-type strain to one of the mutants (Qi et al., 2017). Gene ontology enrichment analysis highlighted distinctions in differential expression of stress response and antioxidant proteins, though the identities of these proteins were not published. Enzymes involved in DNA repair, spliceosome assembly, acetate utilization (e.g., dehydrogenases), and MAPK signaling were upregulated in the mutants. MAPK-related proteins STE20 and SSK2 were highly expressed, perhaps to maintain cell integrity under high-osmolarity hydrolysate cultivation and efficient oxidative phosphorylation by mitigating oxidative stress (Fuchs and Mylonakis, 2009) caused by hydrolysate-derived inhibitors. Interestingly, these proteins are upstream regulators of HOG1 in *S. cerevisiae* (Raitt et al., 2000)—as discussed above, knocking-out HOG1 in *Y. lipolytica* increases lipid production (Poorinmohammad et al., 2022). Intriguingly, its role in stress-induced regulation of lipid homeostasis may ultimately be conserved even in unrelated yeast.

Industrially-relevant oleaginous yeast must rapidly assimilate both hexose and pentose sugars derived from low-cost lignocellusic substrates. *R. toruloides* not only grows slower on xylose than glucose but also exhibits a diauxic shift when switching between these sugars (Wiebe et al., 2012). Coradetti et al. (2023) used proteomics to study diauxic sugar utilization including transport and regulation of carbon catabolite repression, which will help unveil targets to engineer glucose and xylose co-utilizing strains. In their previous study of RB-TDNA mutants discussed above (Coradetti et al., 2018; Kim et al., 2021), mutations in a zinc binuclear cluster transcription factor (RTO4_12978, or PNT1) greatly decreased fitness on pentose sugars. Proteomic analysis of PNT1 overexpression and deletion mutants grown on xylose confirmed the role of PNT1 in regulating xylose assimilation, but also raised some confounding questions regarding PPP regulation (Coradetti et al., 2023). Interestingly, PNT1 overexpression decreases the relative abundance of xylulose phosphoketolase, which breaks down xylulose 5-phosphate to produce acetyl phosphate and glyceraldehyde 3-phosphate. It appears that xylose assimilation regulates how carbon flows through the non-oxidative portion of the PPP—bypassing pyruvate dehydrogenase and the TCA to directly produce acetyl-CoA for fatty acid synthesis or dihydroxyacetone phosphate and other PPP anabolic precursors for phospholipids and nucleosides. Their exemplary work demonstrates how regulons can be deduced from proteomics and strain engineering data, providing unexplored targets for optimizing pentose utilization.

### 4.3 Other yeasts of biotechnological value

#### 4.3.1 Sophorolipid synthesis

**Figure 6:**
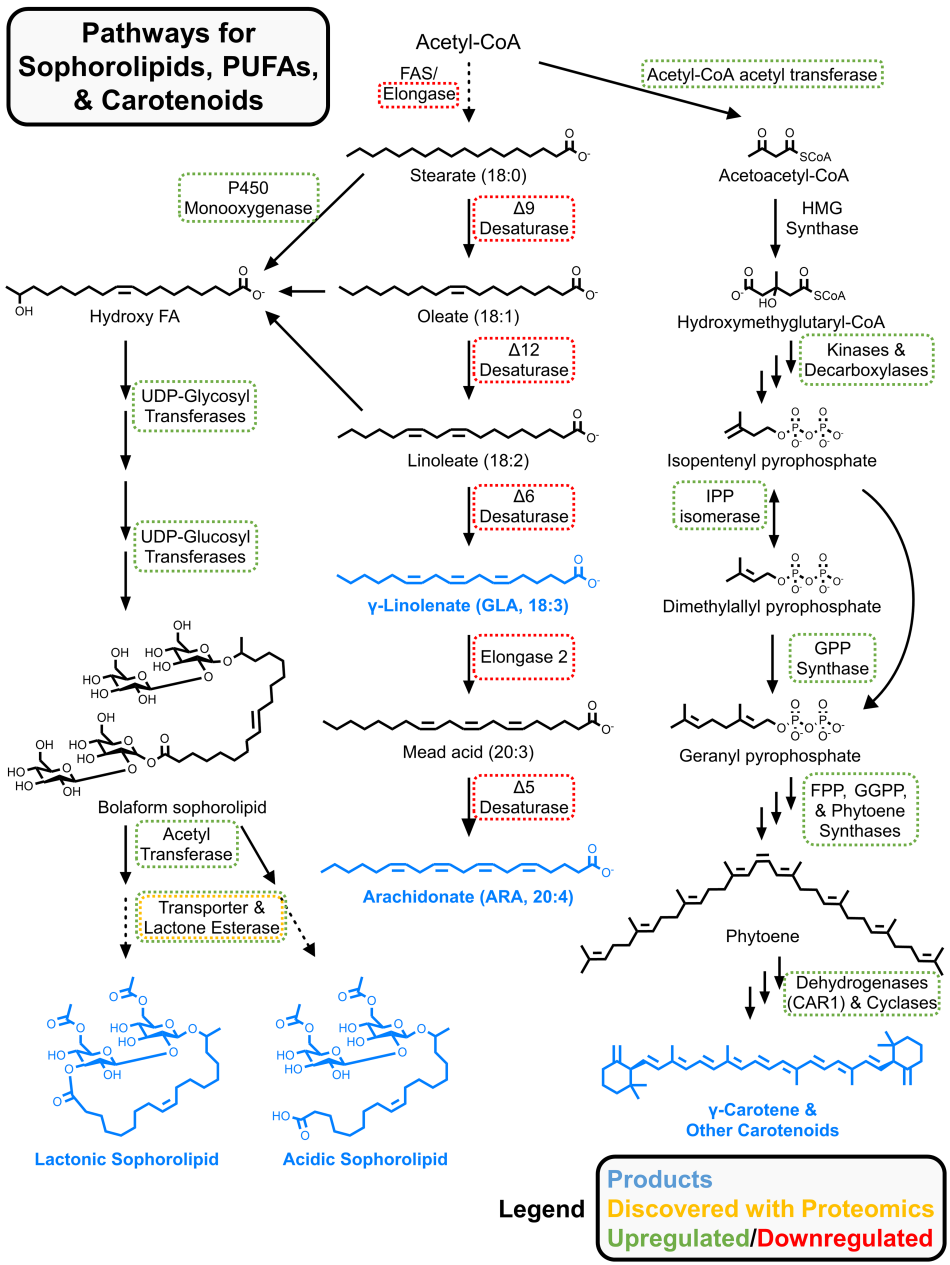
Metabolic pathways for sophorolipids, carotenoids, and omega-3/6 fatty acids. Proteomics results from multiple studies are summarized. Up/downregulated refers to relative changes to protein abundances for nutrient limited vs. rich conditions. Generalized abundance changes were pulled from references in subsection 4.3 (for sophorolipids and carotenoids) and section 5 (for omega-3/6 fatty acids).

Sophorolipids are glycolipid biosurfactants with antimicrobial properties, making them valuable for acne treatments, eco-friendly cleaning solutions, and other food and health applications (Cho et al., 2022). The oleaginous yeast *Starmerella bombicola*, isolated from bumblebees, secretes sophorolipids and is a star candidate for metabolic engineering (Roelants et al., 2024). Ciesielska et al. performed two proteomics investigations of sophorolipid synthesis in *S. bombicola* (Ciesielska et al., 2013, 2014). In the first, they used an auxotrophic strain to perform SILAC-based characterization of exponentially growing cells vs. those producing sophorolipids in stationary phase (Ciesielska et al., 2013). In the first step of sophorolipid biosynthesis, fatty acids are hydroxylated by a membrane-bound cytochrome P450 monooxygenase (Cyp52M1) (Van Bogaert et al., 2009), which was only identified in the stationary growth phase. The authors noted that a heme-binding protein DAP1 was upregulated and may enhance Cyp52M1 activity through stabilization. Other enzymes involved in sophorolipid synthesis like UDP-glucosyltransferases were upregulated (see Figure 6 for full metabolic pathway Roelants et al., 2024). In the stationary phase, oxidative stress defense enzymes and vacuolar proteins were upregulated, while proteins involved in translation were downregulated. In their second secretomics study, Ciesielska et al. (2014) identified an esterase required for the lactonization of secreted sophorolipids and knocked out this enzyme for functional validation, providing another example of how proteomics insights can direct metabolic engineering.

#### 4.3.2 Carotenoid synthesis

The *Rhodotorula* genus includes several carotogenic oleaginous yeast with the characteristic red color, including *R. toruloides*. The mevalonate pathway is used to produce carotenoids from acetyl-CoA. The first step involves acetyl-CoA acetyltransferase to produce acetoacetyl-CoA, which, together with acetyl-CoA, is condensed to form hydroxymethylglutaryl-CoA (HMG-CoA) by the corresponding synthase (Kot et al., 2016; Ochoa-Viñals et al., 2024). Then, HMG-CoA is transformed to mevalonic acid by a specific reductase. Several steps involving a kinase, decarboxylase, isomerase, prenyl transferase, phytoene synthase and desaturase, and cyclases ultimately produce a mix of carotenoids. These include carotenes (*α*-, *β*-, and *γ*-isomers), torulene, and torularhodin (Figure 6).

Although most proteomics studies of oleaginous yeast focus on nitrogen availability, oxygen availability also affects lipid accumulation and carotenoid synthesis (Calvey et al., 2016; Mosqueda-Martínez et al., 2024; Thumkasem et al., 2024). Fakankun et al. employed proteomics to investigate the effects of dual nitrogen and oxygen limitation on lipogenesis and carotenoid production in *R. diobovata* (Fakankun et al., 2021). Samples were analyzed for the late exponential and early stationary phase at which point carotenoid concentrations began to increase. During early stationary phase, upregulation of malic enzyme and PPP enzymes was observed, but an expected increase in lipid biosynthesis was not, indicating a redirection of reducing equivalents away from fatty acid synthesis. Key enzymes in the mevalonate pathway and carotenoid synthesis including acetyl-CoA C-acetyltransferase (also known as thiolase), phosphomevalonate kinase, and phytoene dehydrogenase were upregulated suggesting that acetyl-CoA flux was preferentially directed toward carotenoid biosynthesis. Nevertheless, other studies have shown the importance of oxygen availability for carotenoid production (Wang and Yu, 2009; Thumkasem et al., 2024).

*Xanthophyllomyces dendrorhous* (teleomorph of *Phaffia rhodozyma*) is another oleaginous yeast (Flores-Cotera et al., 2001; Chávez-Cabrera et al., 2010) of biotechnological intrigue for its production of astaxanthin, an antioxidant carotenoid (Martinez-Moya et al., 2011). Martinez-Moya et al. (2015) conducted a proteomic and metabolomic analysis of how this yeast regulates astaxanthin synthesis according to carbon source (glucose or succinate). When succinate is supplied, carotenogenesis is induced during the exponential phase, whereas for glucose it is induced during the stationary phase—demonstrating dependence on not only resource availability over time (e.g., nitrogen) but also carbon source. Their metabolomics results largely follow expectations for carbon source entry points into primary metabolism (glycolysis/PPP for glucose and TCA cycle for succinate). Unfortunately, considerations about how exactly NADPH would be regenerated using succinate (e.g., NADP+ dependent isocitrate dehydrogenase) were not discussed despite its requirement for carotenoid synthesis. Because succinate is transformed into fumarate by succinate dehydrogenase (upregulated in succinate condition; Complex II of the electron transport chain), they propose that respiratory ROS formation is a mechanism by which carotenogenesis is regulated. Though the antioxidant enzyme expression levels do not fully align with this conclusion (e.g., downregulation of peroxiredoxin, TSA2), astaxanthin synthase and cytochrome P450 reductase were significantly upregulated on succinate. This is in addition to phosphoglucomutase, which is involved in trehalose formation and often associated with stress response (Weeks et al., 2006).

#### 4.3.3 Additional important chassis for TAG accumulation

Though most proteomics studies have been conducted on *Y. lipolytica* and *R. toruloides*, these are not the only promising lipogenic chassis for metabolic engineering. *L. starkeyi* and *Cutaneotrichosporon oleaginosum* (formerly *Crytococcus curvatus* and *Trichosporon oleaginosus*) are considered top lipid producers, with both naturally utilizing and in some cases co-utilizing (Gong et al., 2012; Yang et al., 2014; Yu et al., 2014) a variety of biomass-derived carbon sources. Studies using these two yeasts are reviewed in this subsection.

Liu et al. (2011) performed the first timecourse proteomics investigations of oleaginous yeast, including one in which *L. starkeyi* was cultivated in nitrogen-rich vs. deficient media with a high concentration of glucose (Table 2). Many of the primary metabolic processes (e.g., alternative nitrogen source utilization and protein, amino acid, and nucleic acid turnover) discussed in the previous subsections exhibited changes. Increased lipid production was linked to upregulation of key TCA and transhydrogenase cycle enzymes, including citrate synthase (CIT1) to buildup citrate and route carbon towards fatty acid biosynthesis. Acetyl-CoA carboxylases ACC1 (cytosolic) and HFA1 (mitochondrial) were also upregulated in the late culture stage. Additionally, the drastic upregulation of 6-phosphogluconate dehydrogenase (GND2) provides early support for the PPP’s role in supplying NADPH for fatty acid biosynthesis. With this and their earlier study (Liu et al., 2009)—as well as other endeavoring to study protein expression in unconventional microorganisms--it’s evident that having a sequenced and well-annotated genome is crucial for maximizing proteomics’ potential.

Two proteomics research areas related to carbon efficient bioconversion of biomass hydrolysates that have largely been shirked were studied by the Blenner and Brueck labs using *C. oleaginosum*: lignin-derived aromatics utilization (Yaguchi et al., 2017b; Yaguchi, 2020) and carbohydrate-active enzymes (CAZy) for transport and metabolism of mono-, and importantly, disaccharides (Fuchs et al., 2021). Yaguchi harnessed multi-omics and biochemical assays to elucidate catabolic pathways for monoaromatics including phenol, resorcinol, p-hydroxybenzoate, ferulate, and *p*-coumarate, as well as the yeast’s growth on an alkaline-pretreated corn stover rich in lignin, aromatics, and acetate (Yaguchi, 2020). Yaguchi annotated several transporters, stress response proteins, and metabolic enzymes such as dioxygenases, which perform the ring-cleaving step of aromatics degradation. Fuchs et al. performed a comprehensive proteomics analysis of secreted, cell wall-associated, and cytoplasmatic proteins to uncover enzymes (based on EC, enzyme classes) that cleave and transport dissaccharides (Fuchs et al., 2021). These include various glycoside hydrolases (GH) found in different CAZy database (sub)families; however, it’s unclear if the CAZy database (Levasseur et al., 2013) was specifically used to search their results. Nonetheless, compared to a glucose control, significant differences in the cell-bound and secreted fractions were observed for *α*-galactosidase, *β*-galactosidase, β-glucosidase, β-mannosidase and α-amylase as expected under carbon catabolite derepression.

## 5 Proteomics insights for filamentous fungi

Though fewer compared to studies of oleaginous yeast, proteomics investigations of oleaginous filamentous fungi have revealed important insights regarding stress response and metabolic regulation during nutrient limitation. *Mucor circinelloides* and *Mortierella alpina* have been used in these studies due to their ability to produce omega-3 and omega-6 fatty acids (Zhang X. et al., 2022). In addition to accumulating TAGs in media with high C: N ratios, *M. circinelloides* has been primarily investigated for native synthesis of β-carotene and significant quantities of *γ*-linolenic acid (GLA; *ω*-6 C18:3) (Fazili et al., 2022b; Wang et al., 2022). It has also been engineered to produce the skin and neurological health-promoting antioxidant astaxanthin from *β*-carotene (Papp et al., 2006; Hameed et al., 2017; Zhou et al., 2021; Adıgüzel and Ülger, 2024). Moreover, *M. circinelloides* secretes cellulases and hemicellulases to improve carbon utilization from pretreated lignocellulose and potentially reduce the cost of enzymatic saccharification (Wei et al., 2013; Al Mousa et al., 2022a,b).

*Mortierella alpina* is popular for its ability to produce arachidonic acid (ARA; *ω*-6 C20:4), and some strains even produce eicosapentaenoic acid (EPA; *ω*-6 20:5) among other economically attractive, bioactive PUFAs (Yamada et al., 1987; Shimiziu et al., 1988; Singh and Ward, 1997; Takeno et al., 2005; Kikukawa et al., 2018; Chang et al., 2022). It is generally regarded as safe with FDA approval for use of its ARA-rich oil in infant formulation (Ratledge et al., 2010; Ryan et al., 2010). For these reasons, proteomics investigations of oleaginous filamentous fungi have focused exclusively on these particular species (Table 2). Overall, these studies highlight the intricate role of autophagy and alterations to central carbon metabolism that are crucial for understanding lipid accumulation.

**Table 2:** Summary for proteomics studies of oleaginous filamentous fungi.

Approach	Multi-omics	Stress	Organism	Carbon	Nitrogen	Experimental	Lipid titer/content	IDs	Significant findings summary	Ref.
Label-free lipid droplet and cellular proteomics	-	Carbon	*M. alpina*	80 g/L Glucose	YE NaNO3	Timecourse (156 and 192 h); Batch flasks	~11 g/L, ~40%; 10 g/L, ~38%	> 411^‡,§^	Fructose metabolic enzymes associated with lipid droplets. Hydratase in PUFA synthesis upregulated during aging.	Yu et al. (2016, 2017)
Label-free lipid droplet and cellular proteomics	M	Carbon	*M. alpina*	80 g/L Glucose	KNO3 Urea	192 h; Control vs. nitrogen source supplemented during aging; Batch	12.7 g/L, ~49% (6.48 g/L ARA; max using KNO3)	> 500^‡,§^	KNO3 stimulated pyruvate-malate cycle and PPP. Antioxidants associated with and differentially regulated in LD proteome.	Yu et al. (2018)
Label-free cellular proteomics	M, L	Nitrogen	*M. alpina*	30 g/L Glucose	Diammonium tartrate YE	Timecourse (many time points); Batch fermentation tanks	~2.9 g/L, ~32% max	3,462	Resource reallocation contributes to TAG increase not abundance changes. Explored ubiquitin-mediated proteolysis, ER-associated degradation, and unfold protein response.	Lu et al. (2020)
Selective (MALDI-TOF-MS/MS)	-	Nitrogen	*M. alpina* *C. echinulata* *Schizochytrium*	Varied glucose concentrations	Varied	Timecourse (varying time points for each organism); Batch flasks	See manuscript	12^§^	Homogenization + cold shock is a cheap, optimal method for lipid removal. HSP90 and desaturases upregulated in fungi.	Ling et al. (2015)
Selective (MALDI-TOF-MS/MS)	-	-	*M. circinelloides*	PDA Cheese-mimicking medium	YE Protein mix	168 h; M. circinelloides (cheese contaminant) compared to other fungi; Batch plates	-	494^§^	Proteasome, glycogen mobilization, lipid metabolism, purine degradation, and stress response upregulated in M. circinelloideson cheeese mimic.	Morin-Sardin et al. (2017)
Label-free Secretomics	-	Carbon	*M. circinelloides*	10 g/L Lactose	YE (NH4)2SO4	120 h; Qualitative investigation; Batch flasks	-	25^§^	Combination of secretome extraction methods boosted coverage. Endoglucanases, polysaccharide deacetylase, and β-glucosidase observed.	Wei et al. (2013)
Selective (MALDI-TOF/TOF-MS)	-	Nitrogen	*M. circinelloides*	80 g/L Glucose	Diammonium tartrate YE	24 h; High vs. low-lipid producing strains; Batch fermenters	~2.5 g/L, ~21%; ~0.8 g/L, ~9%	> 800^‡.§^	Enzymes for acetyl-CoA (e.g., from lysine), NADPH, and nitrogen metabolism upregulated in high-lipid strain.	Tang et al. (2017)
Selective (MALDI-TOF-MS/MS)	-	Nitrogen	*M. circinelloides*	80 g/L Glucose	Diammonium tartrate YE	Timecourse (6, 24, and 60 h); Batch fermentors	~4.3 g/L, ~33%	> 800^‡,§^	Glycolysis and PPP upregulated while TCA was downregulated. No change in ME expression. Antioxidants and glutathione metabolisc enzymes upregulated.	Tang et al. (2016)

Refer to legend below Table 1.

### 5.1 Mucor circinelloides

#### 5.1.1 γ-linolenic acid production

The oleaginous *M. circinelloides* strain WJ11 was isolated and sequenced by Tang et al. Under nitrogen limitation, this strain accumulates ≥ 36% of its CDW as lipids with a ~ 13% GLA content (Tang et al., 2015a, 2015b). GLA is produced via multiple desaturation reactions: stearic acid (C18:0) is converted to oleic acid (C18:1) by Δ9-desaturase and further desaturated to linoleic acid (C18:2) by Δ12-desaturase and, finally, to GLA by Δ6-desaturase (Figure 6) (Zhang Y. et al., 2017). Two proteomics studies were conducted on *M. circinelloides* WJ11 (Table 1): a time-course experiment in which cultures were sampled at 6 h (exponential phase), 24 h (rapid lipid production phase), and 60 h (stationary phase) (Tang et al., 2016) as well as an experiment comparing *M. circinelloides* WJ11 to strain CBS 277.49, a low lipid but high carotenoid-producing strain (Tang et al., 2017). In both studies, a modified K & R growth medium (Kendrick and Ratledge, 1992) was used, which contained 80 g/L glucose, 2 g/L diammonium tartrate, 1.5 g/L yeast extract (~80:1 C: N ratio), and other defined nutrients.

In the timecourse experiment, nitrogen limitation generally led to downregulation of the TCA cycle and amino acid biosynthesis (Tang et al., 2016); however, *S*-adenosylmethionine synthase, which participates in methionine degradation, was upregulated and may provide resources for glutathione synthesis. In a later study using genome-scale metabolic modeling and data integration, methionine degradation was highlighted and recapitulates the importance of sulfur amino acid metabolism during nitrogen-limited stress response (Vongsangnak et al., 2018). In further support of the role of antioxidants during nitrogen limitation, *S*-formylglutathione hydrolase was upregulated and hydrolyzes *S*-formylglutathione to restore glutathione that had condensed with formaldehyde. Amino acid degradation, demethylation reactions, and tetrahydrofolate (THF)-dependent pathways can generate formaldehyde (Weimer et al., 1993; Degrassi et al., 1999; Pham et al., 2023). Interestingly, they observed upregulation of the antioxidants peroxiredoxin and glutathione peroxidase, which detoxify peroxides (Pócsi et al., 2004). Strengthening the link between antioxidants and the oleaginous phenotype, peroxiredoxin and catalase were upregulated in strain WJ11 compared to strain CBS 499.25 during nitrogen limitation (Tang et al., 2017). A thiazole biosynthetic enzyme involved in thiamine metabolism (also upregulated in WJ11 compared to CBS 499.25), a 14–3-3 family protein that promotes cell survival by negatively regulating apoptosis, and heat shock proteins for regulating proper protein folding were all upregulated (Tang et al., 2016). These present interesting yet unexplored targets for engineering improved stress response.

Concurrently, pyruvate kinase, glyceraldehyde-3-phosphate dehydrogenase, fructose bisphosphate aldolase, and enolase were upregulated in the timecourse experiment of WJ11; these enzymes function at the intersection of the PPP, glycolysis, and lipid synthesis. For instance, enzymes in the PPP were upregulated to generate NADPH—a relationship that had been established prior to proteomics(Zhao et al., 2015; Masi et al., 2021). Nevertheless, glucose-6-phosphate dehydrogenase was upregulated compared to the lower lipid-producing strain CBS 477.25, reinforcing the role of PPP for the oleaginocity (Tang et al., 2017). The expression of ME did not change in both the timecourse experiment and comparative proteomics study (Tang et al., 2016, 2017); however, *M. circinelloides* contains several isoforms of ME with only one being NADP^+^-dependent (Song et al., 2001). Because the full set of proteomics data was not published and this enzyme was not listed in the table of presented results, it is unclear which isoform they are referring to (Tang et al., 2016). Moreover, studies of ME overexpression in different strains are conflicting (Zhang et al., 2007; Rodríguez-Frómeta et al., 2013; Fazili et al., 2022a). Two additional insights related to acetyl-CoA flux were gleaned from the comparative proteomics study. Firstly, aldehyde can be produced from pyruvate and converted to acetate by aldehyde dehydrogenase, thereby providing an alternative source of acetyl-CoA: this enzyme was upregulated in WJ11 compared to CBS 277.49. Secondly, degradation of branched-chain amino acids (e.g., leucine) and lysine provides acetyl-CoA: enzymes that degrade these amino acids were upregulated in WJ11 (Tang et al., 2017). As yet another example of proteomics results directing subsequent metabolic engineering studies, Tang et al. overexpressed glucose 6-phosphate dehydrogenase and *β*-isopropylmalate dehydrogenase in CBS 277.49 leading to as much as a 47 and 73% increase in lipid content, respectively (Tang et al., 2020).

### 5.2 Mortierella alpina

#### 5.2.1 Arachidonic acid production

In contrast to the conditions used for lipid production in *M. circinelloides*, ARA production using *M. alpina* is often conducted via a multistage aging process, during which biomass is first generated in a nutrient-replete environment, followed by a transition (or even transfer of mycelia) to a carbon-deficient environment (Ji et al., 2014). Jin et al. optimized a fed-batch strategy using strain ME-1 to yield as much as 19.8 g/L ARA (Jin et al., 2008). Overall, this process is characterized by long cultivation periods, and proteomics sampling was conducted at extended (~200 h) time points (Table 1). Three similar studies by Yu et al. harnessed this strategy to produce appreciable quantities of ARA (~ 6 g/L) via batch cultivation of strain R807 on 80 g/L glucose, 3 g/L NaNO_3_, and 10 g/L yeast extract (~20:1 C: N ratio), and other nutrients (Yu et al., 2016, 2017, 2018). In their first study, label-free proteomics was used to study the change of cellular proteins during aging of *M. alpina* for ARA accumulation, revealing a total of 171 significant proteins that are enriched in ROS response (Yu et al., 2016). Next, the same group quantified changes in the lipid droplet proteome of *M. alpina* during aging, identifying significant changes in 62 out of the 400 lipid droplet-associated proteins (Yu et al., 2017). In a more recent study, a similar analysis of the lipid droplet proteome, as well as the cellular proteome, was performed to study the impact of two nitrogen sources (KNO_3_ and urea) on ARA production (Yu et al., 2018).

Comparing a nutrient-rich time point to a later time point during carbon starvation, their proteomics results showed a characteristic shift towards autophagy: enzymes involved in amino acid degradation were upregulated in concert with a minor increase in several amino acids suggesting protein turnover (Yu et al., 2016). An uncharacterized autophagy-related protein and Rab GTPases were observed in the lipid droplet proteome underlining the role of lipid droplet in lipid and protein trafficking (Martin and Parton, 2008; Welte, 2015). As expected, carbohydrate catabolic pathways and fatty acid biosynthesis (e.g., ACC) were also generally suppressed while the PPP exhibited insignificant changes in this study (Yu et al., 2016). Interestingly, a relative increase in PPP and fatty acid biosynthesis enzymes was found when KNO_3_ was supplemented during aging, which may contribute to the slight though significant increase in ARA (Yu et al., 2018).

During carbon starvation, lipids from the lipid droplets can be mobilized via *β*-oxidation to provide ATP and acetyl-CoA for TCA cycle anaplerosis (Enkler and Spang, 2024). Accordingly, Yu et al. (2017) observed aggregates of enlarged mitochondria and shrunken lipid droplets, correlating with upregulation of some β-oxidation enzymes (e.g., acetyl-coA synthetase). During aging, two enzymes involved in fructose, sucrose, mannose, and fucose metabolism were upregulated and localized to lipid droplets, supporting an autophagic or energy balancing role of lipid droplets in antioxidant ascorbate metabolism, cell wall remodeling, or recycling amino/nucleotide sugars, glycoproteins, and/or sucrose stores that accumulated during growth (Yu et al., 2017, 2018). Interestingly, one of these enzymes is GDP-keto-6-deoxymannose 3,5-epimerase/4-reductase that uses either NADH or NADPH (Ren et al., 2010). In the cellular proteome, the abundances and measured enzymatic activities for NADP^+^-dependent ME decreased while ICDH increased, suggesting a repressed transhydrogenase cycle and activity of an alternative cytoplasmic NADPH-generating shuttle to supply reducing power for fatty acid biosynthesis and antioxidant defense (Tang et al., 2021). Though a thorough explanation is absent, it’s curious that glutathione metabolism was significantly downregulated according to KEGG pathway analysis, and that they noted a drastic increase in ROS during aging according to an assay using a fluorogenic probe (Yu et al., 2017).

#### 5.2.2 Nitrogen limitation-induced resource reallocation

Lu et al. investigated the response of *M. alpina* to nitrogen limitation using an integrated lipidomics, metabolomics, and proteomics approach (Lu et al., 2020). They cultured *M. alpina* strain ATCC 32222 under nitrogen limitation with a medium containing 30.0 g/L glucose, 2.0 g/L diammonium tartrate, and 1.5 g/L yeast extract (~30 C: N ratio) and observed a drop in ARA and TAG accumulation. Enzymes and the corresponding glycolytic metabolites were downregulated during nitrogen limitation, whereas TCA cycle enzymes were generally downregulated with a concomitant accumulation of citrate. ME was downregulated while ACL and phosphoenolpyruvate carboxykinase were upregulated, providing a route from citrate to phosphoenolpyruvate to feed gluconeogenesis. Though they do not explicitly report the expression levels of FAS1/2 nor ACC, they conclude that TAG accumulation was a consequence of resource reallocation, rather than an upregulation of fatty acid biosynthesis enzymes. This is a reasonable conclusion given that succinate increased up to the 144 h time point, paralleling an increase in succinate-semialdehyde dehydrogenase and glutamate decarboxylase of the GABA shunt, which provides a TCA cycle entry point for autophagy-derived carbon. According to their lipidomics and proteomics results, an increase in TAGs may be additionally explained by upregulation of phospholipases C and D, which convert phosphatidylcholine and phosphatidylethanolamine into precursor DAGs.

The relative quantities of proteins, peptides, amino acids, and a plethora of other nitrogenous compounds decreased during nitrogen limitation (Lu et al., 2020). This is corroborated by an increase in ubiquitin-proteasome system components, including 26S proteasome subunits and ubiquitin-activating enzyme E1 during the early stages of nitrogen deprivation. The importance of autophagic processes are further highlighted by an increase in autophagy-related proteins, vacuolar components, and peroxisomal enzymes—including a catalase that detoxifies H_2_O_2_ generated as a byproduct of β-oxidation, which was upregulated during nitrogen limitation. Autophagic processes are negatively regulated by TOR (Wang et al., 2023b) in nutrient-rich environments: in their results, a homolog of mammalian TOR decreased in abundance over time. A regulatory link between autophagy and lipid metabolism has been established but little is known in oleaginous filamentous fungi (Singh et al., 2009). Excitingly, in a follow-up study, they harnessed their proteomics insights to delve into autophagy-regulated lipid metabolism: they showed that overexpression of autophagy-related gene 8 (ATG8) increased fatty acid biosynthesis by ~10% (Lu et al., 2022).

## 6 Cross-species patterns in proteomic signatures of lipogenesis

Across species like *Yarrowia lipolytica*, *Rhodotorula toruloides*, and *Mucor circinelloides*, differential expression of key lipid biosynthesis enzymes—ATP citrate lyase (ACL), acetyl-CoA carboxylase (ACC), and/or fatty acid synthase (FAS)—is a common signature under nutrient limitation (Zhu et al., 2012; Pomraning et al., 2016; Tang et al., 2017). Lipid droplet-associated proteins, such as the perilipin-like proteins OIL1 in *Y. lipolytica* (Bhutada et al., 2018) and LDP1 in *R. toruloides* (Zhu et al., 2015), are consistently upregulated, suggesting a conserved role in regulating lipid droplets and storage lipids. Differential expression of autophagy-related proteins, including ATG8 and ATG9 which are discussed in studies of *R. toruloides* (Wang et al., 2023a) and *Mortierella alpina* (Lu et al., 2020), also suggests resource recycling as a universal stress response to nutrient limitation. In addition, some components of autophagy-regulated lipid metabolism are conserved (Wang et al., 2023b), including the AMP deaminase-isocitrate dehydrogenase (ICDH)-citrate axis that reroutes carbon to lipid biosynthesis under nitrogen limitation. Phosphoregulation of ACL and ACC is another shared feature, though specific phosphorylation sites may differ (Pomraning et al., 2016; Wang et al., 2023a).

The pentose phosphate pathway (PPP) is a major source of NADPH across fungi. There are additional species-specific sources like NADP^+^-dependent ICDH in *Y. lipolytica* and malic enzyme in *R. toruloides* and *M. circinelloides* (Ratledge, 2014). Of course, differing NADPH requirements and regeneration reactions may explain differences in protein expression for the corresponding pathways in central carbon metabolism. However, this is difficult to evaluate without a detailed phyloproteomics investigation or, preferably, direct comparisons within sample sets analyzed using the same proteomics workflow. In addition to fatty acid synthesis, NADPH is required for antioxidant defense against pro-oxidants such as hydrogen peroxide. This and other reactive oxygen species are readily generated during respiration, as a byproduct of purine and amino acid scavenging, as well as β-oxidation (Sibirny, 2016; Picazo and Molin, 2021; Mattila et al., 2022). Though the number and types of antioxidant proteins differ among fungi (Mattila et al., 2022), a link between starvation stress response and redox systems was pronounced for many of the studies reviewed herein. In a recent publication, a multi-PTM proteomics and lipidomics approach was used to reveal that nitrogen limitation in *R. toruloides* drives widespread changes in protein thiol oxidation and phosphorylation for stress response signaling, metabolic pathways, and autophagy (Gluth et al., 2025).

## 7 Discussion

MS-based proteomics can quantify thousands of proteins (and modifications to them) in parallel, thereby revealing key cellular pathways that culminate in a specific phenotype. In this review, we demonstrate the power of proteomics for characterizing molecular events that drive oleochemical production in oleaginous fungi. Applications of proteomics in oleaginous fungi in the last decade has pointed to the role of multiple pathways/processes governing oleaginicity including signaling pathways (TOR, AMPK, MAPK, etc.), redox balance (including antioxidant defense), autophagy, nitrogen metabolism (especially pertaining to branched chain amino acids, asparagine and the urea cycle, and purine degradation), transport, cellular trafficking, and energy homeostasis. Comparative proteomics has also identified proteins that can be used as engineering targets to improve lipid content. In addition, subcellular proteomics of lipid droplets has revealed this organelle’s multifaceted role in energy and redox homeostasis as well as stress response (Jarc and Petan, 2019)—even identifying the associated proteins that regulate access to its carbon stores (Zhu et al., 2015; Bhutada et al., 2018).

Even though this review is comprehensive, we were challenged by the fact that oleaginocity is generally strain-dependent (Salvador López et al., 2022), may involve nuances that are not completely conserved or well-annotated, is not represented solely by storage lipids (TAGs) as the primary oleochemical product, and is not always the focus of proteomics investigations. There are studies of oleaginous fungi that were not included here because (1) “oleaginous” was not explicitly mentioned in the study (Martinez-Moya et al., 2020), (2) conditions conducive to lipid production were not studied or oleochemicals were not the focus (Morín et al., 2007; Mansour et al., 2009; Swennen et al., 2010; Navarro et al., 2013; Celińska et al., 2015; Yang et al., 2015; Zhang H. et al., 2017; da Silva et al., 2020; Sekova et al., 2021; Li X. et al., 2022), and/or (3) it is unclear if the strain in question is indeed oleaginous. For instance, some strains of *Aspergillus* and *Geotrichum* species are oleaginous, yet their proteomes have not been investigated for lipogenic conditions (Thammarongtham et al., 2018; Grygier et al., 2019; Srinivasan et al., 2022; Kamilari et al., 2023; Hassane et al., 2024). Another example is the yeast *Debaryomyces hansenii*, which has been studied for its halophilic behavior and could potentially reduce the demand for fresh water during lipid production (Yaguchi et al., 2017a; Navarrete et al., 2022).

The proteomics field is still progressing along with the appropriate and relevant applications. Proteomics data sharing is of great importance for cross-study comparisons and is gradually improving (Shome et al., 2024); however, some of the studies reviewed herein do not have accompanying (or well-annotated and organized) datasets, which severely mars the interpretation of results and stymies a phyloproteomics investigation of carbon storage evolution in fungi. Moreover, some do not explicitly include details for their bioinformatics approaches or erroneously compare intensity values among different proteins despite not using an approach for absolute quantification (Ankney et al., 2018). This raises an intriguing opportunity, though: targeted proteomics, in which libraries of peptide standards are used for absolute quantification of proteins, has also not been applied to oleaginous fungi. Overall, integrating proteomics data into genome-scale metabolic models (GEMs) will be essential for predictive design, and future work should prioritize absolute quantification to support accurate kinetic modeling. Quantitative proteomics can refine enzyme-constrained models and improve flux balance predictions (Tang et al., 2017; Kim et al., 2021). Furthermore, incorporating absolute enzyme quantities with metabolomics data would provide valuable inputs to construct highly accurate metabolic models and engineer optimal lipid production (Yunus and Lee, 2022). Artificial Intelligence / Machine Learning (AI/ML) can analyze large proteomic datasets to predict protein functions, interactions, and regulatory networks (Li F. et al., 2022; Smith et al., 2022). For oleaginous fungi, AI/ML could identify novel lipid biosynthesis regulators by integrating proteomics with transcriptomics and metabolomics (Ballard et al., 2024).

Several newer proteomics strategies have yet to be explored in oleaginous fungi but could significantly improve the scalability needed for large-scale studies and the resolution required for investigations that, for example, aim to characterize how different populations of yeast cells respond to the microenvironments which are present even in well-mixed bioreactors (Boswell et al., 2003). Regarding the former, data-independent acquisition (DIA) is an emerging technique for label-free quantification and has yet to be applied for proteomics investigations of oleaginous fungi, even though cost savings could be realized by avoiding expensive mass tags and the throughput of modern LC–MS/MS systems has been rapidly improving. Single-cell proteomics is another emerging frontier in systems biology that will enable dissection of cell-to-cell heterogeneity in lipid accumulation (Shuken, 2023), particularly relevant for filamentous fungi, where hyphal differentiation may lead to varied lipogenic capacities within a culture. This approach could uncover cell-specific proteomic signatures in *Y. lipolytica* or *R. toruloides* under nutrient limitation, identifying subpopulations optimized for lipid accumulation. For example, single-cell proteomics could elucidate why certain cells within a culture exhibit higher TAG storage. Techniques such as SCoPE-MS and NanoPOTS offer new possibilities for such investigations (Budnik et al., 2018; Zhu et al., 2018; Martin et al., 2021).

Looking forward, the burgeoning application of proteomics in synthetic biology is rife with opportunity. Frequently, the rationale for single-cell oil production is tied to the valorization of low-cost organic wastes, especially lignocellulosic feedstocks. However, there are surprisingly few proteomics studies of the fungal oleaginous phenotype using these complex substrates and scaled-up cultivations (> 5 L). Thus, one direction for future studies would be the analysis of dynamic carbon utilization under bioprocessing-relevant scenarios with engineered vs. wildtype strains. There are currently no metaproteomics studies of oleaginous fungi co-cultured with carbon- and/or nitrogen-fixing microorganisms (algae, cyanobacteria, azitobacter, etc.) (Bohutskyi et al., 2024). Additionally, the few quantitative proteomics investigations of PTMs have focused exclusively on phosphorylation, even though methylation, acetylation, ubiquitination, and cysteine oxidation (redox proteomics) also regulate protein function, proteasomal processing, signaling pathways, and transcriptional programs (Tripodi et al., 2015; Šoštarić and van Noort, 2021). For instance, the OIL1 protein of *Y. lipolytica* protects the lipid droplet from being accessed by lipases—it is ubiquitinated (Bhutada et al., 2018), raising the question of how much oleaginocity is regulated by the PTMome vs. changes to enzyme abundances. Indeed, this conclusion was reached in a recent publication that describes a novel proteomics approach for profiling multiple types of PTMs (Table 2), specifically protein cysteine oxidation and phosphorylation (Gluth et al., 2025). Protein structure confers function; thus, with sophisticated algorithms to link PTM dynamics to protein structural changes at the proteome level, one can envision substantial improvements to metabolic modeling approaches (i.e., more accurate predictions of k_cat_ values) (Li F. et al., 2022; Smith et al., 2022).

Finally, we recommend integrating multi-omics approaches into the field of oleochemicals production and encourage multi-institutional collaborations to actualize this goal in the near future. It is imperative that we understand how genetic/metabolic engineering affects biological systems as a whole—especially during the multi-phase, stress-induced fermentations that are applied for oleochemicals production. Employing automated workflows (Gluth et al., 2024, 2025) to screen various engineered and wildtype strains over time and in different conditions will provide a wealth of phenotypic data (i.e., growth, lipid production, etc.) that can be paired with proteomics (including PTMs), transcriptomics, metabolomics, and lipidomics data. Together, these data will facilitate the training of AI/ML models to help us connect phenotypes with the underlying molecular mechanisms. This data can then be leveraged to guide genetic/metabolic engineering strategies for targeted phenotypes (i.e., lipid overproduction). The era defined by integrative multi-omics modeling approaches to direct unintuitive metabolic engineering ventures is upon us.
