# Supplementary material for: Proteomic insights into the physiology and metabolism of oleaginous yeasts and filamentous fungi
Source: Front Microbiol. 2025 Sep 5;16:1637123. doi: 10.3389/fmicb.2025.1637123 (PMC12446351; doi:10.3389/fmicb.2025.1637123)
Supplement: Supplementary file 1 [file Table_1.docx]

**Supplementary Table 1.** Summary for proteomics studies of oleaginous yeast.

| **Approach** | **Multi-omics** | **Stress** | **Organism** | **Carbon** | **Nitrogen** | **Experimental** | **Lipid Titer/Content** | **IDs** | **Significant Findings Summary** | **Ref.** |
| --- | --- | --- | --- | --- | --- | --- | --- | --- | --- | --- |
| Label-free Secretomics and Cellular Proteomics | - | Nitrogen | *C. oleaginosum* | 20 g/L Oligo-sugars | YNB (NH_4_)_2_SO_4_ | 48h; Conditions using different sugars compared; Batch flasks | Various; Highest 29.1% lactose; Lowest 9.5% trehalose | 4,498 | Identified hydrolases (e.g., ß-glucosidases) for dimeric sugars and their general localization (secreted, cell wall/membrane-associated, and cytoplasmic). | Fuchs et al. (2021) |
| Label-free Cellular Proteomics | - | Nitrogen | *C. oleaginosum* | 40 g/L Glucose | YE | 8 lysis methods, 13 extraction buffers, and 17 purification methods; Batch flasks | - | 839 | Most IDs observed using homogenization (French press) followed by protein extraction using Urea/Thiourea and C7BzO and TCA/acetone precipitation. | Awad and Brueck (2020) |
| Label-free Cellular Proteomics | G,T | Carbon | *C. oleaginosum* | Pretreated  corn stover 1 g/L Aromatics 1 g/L Glucose | YE (NH_4_)_2_SO_4_ | Carbon source conditions compared (mid-exponential phase cells); Batch flasks | 0.24 g/L, 31.8% for alkaline-pretreated corn stover w/o (NH_4_)_2_SO_4_ | - | First proteomics investigation of lignin-derived aromatics catabolism in oleaginous yeast. Enzymes of beta-ketoadipate pathway annotated. | Yaguchi (2020) |
| Label-free Cellular Proteomics | - | Nitrogen | *L. starkeyi* | 70 g/L Glucose | YE (NH_4_)_2_SO_4_ | Timecourse (8, 48, and 96h); Batch bioreactor | 0.5 g/L, 14%; 12.3 g/L, 28%; 30.0 g/L, 46% | > 250 | Early proteomics results demonstrating upregulation of enzymes in PPP for regenerating NADPH and routing TCA derived carbon to fatty acid biosynthesis. | Lui et al. (2011) |
| Multiplexed Cellular Proteomics | - | Nitrogen Oxygen | *R. diobovata* | ??? g/L Glucose | YE | Timecourse (36 and 48h); Batch flasks | 0.09 g/L, 12.3%; 0.10 g/L 4.6% | 4,408 | Combined oxygen and nitrogen limitation appears to upregulate enzymes for carotenoid synthesis. | Fakankun et al. (2021) |
| Multiplexed Cellular Proteomics | - | - | *R. toruloides* | 40 g/L Xylose | YNB (NH_4_)_2_SO_4_ | Mutant strains comparison; Batch microtiter plate | 1040  mg/L Mutant vs. 460  mg/L Wt, Fatty Alcohols | 4,582 | PNT1 is major transcriptional regulator of pentose metabolism. Glucose transceptor deletion improved xylose utilization. | Coradetti et al. (2023) |
| Multiplexed Cellular Proteomics | L,M,T | - | *R. toruloides* | 10 g/L Glucose 10 g/L Xylose 10 g/L Arabinose  10 g/L Coumarate | YNB (NH_4_)_2_SO_4_ AA Mix | Timecourse (varying time points for each carbon source); Batch flasks | - | 5,643 | Multi-omics data used to improve the metabolic model for lignocellulose-derived carbon sources (xylose, arabinose, and p-coumarate). | Kim et al. (2021) |
| Multiplexed Cellular Proteomics | - | Nitrogen | *R. toruloides C. albidus S. cerevisiae* | 35 g/L Glucose | YE (NH_4_)_2_SO_4_ | Timecourse (varying time points for each organism); Batch flasks | 3.0 g/L, 27.7%; 4.1 g/L, 45.0%;  *S. cerevisiae* not reported | > 300^‡^ | GND1, HSP70/90, GDH3 (NADP^+^), and thioredoxin upregulated in oleaginous yeast vs. *S. cerevisiae* during N limitation. | Shi et al. (2013) |
| Multiplexed Cellular Proteomics | T | Nitrogen | *R. toruloides* | Sugarcane bagasse hydrolysate | YE (NH_4_)_2_SO_4_ | iTRAQ; Timecourse (12, 48, and 96h); WT vs. mutant strain; Batch flasks | - | 4,081 | In hydrolysate-tolerant strain, TCA and glycolytic enzymes upregulated. STE20 of MAPK signaling was also upregulated. | Qi et al. (2017) |
| Label-free Cellular Proteomics | - | Nitrogen | *R. toruloides* | 70 g/L Glucose | YE (NH_4_)_2_SO_4_ | Timecourse (Seed†, 24 and 96h); Batch bioreactor | 0.44 g/L, 9.8%; 3.0 g/L, 37%; 10.7 g/L, 63% | 184 | Effective sample processing workflow established. Nitrogen scavenging and lipid biosynthesis upregulated between rich undefined and defined media. | Lui et al. (2009) |

| **Approach** | **Multi-omics** | **Stress** | **Organism** | **Carbon** | **Nitrogen** | **Experimental** | **Lipid Titer/Content** | **IDs** | **Significant Findings Summary** | **Ref.** |  |
| --- | --- | --- | --- | --- | --- | --- | --- | --- | --- | --- | --- |
| Multiplexed Cellular Proteomics | M | Nitrogen | *R. toruloides* | Corn stover hydrolysate | (NH_4_)_2_SO_4_ Urea | Timecourse (72 and 120h); C:N ratios compared; Batch microtiter plate | Hydroxypropionic acid titers not provided for proteomics experiment | 4,793 | Malonate-semialdehyde dehydrogenase upregulated in condition with highest production and was good target for knockout. | Lui et al. (2023) |  |
| Absolute Cellular Proteomics | - | Nitrogen | *R. toruloides* | 63.6 g/L Glucose 70 g/L Xylose 20.0 g/L Acetate | (NH_4_)_2_SO_4_ Urea | Timecourse (times vary for carbon sources); Batch bioreactors | ~9.6 g/L, 48% glucose; ~3.9 g/L, 30% xylose; ~0.7 g/L, 34% acetate | 3,165 | Flux through PPP and phosphoketolase for acetyl-CoA production increased in nitrogen limitation. | Reķēna et al. (2023) |  |
| Absolute Cellular Proteomics | - | NItrogen | *R. toruloides* | 70 g/L Xylose | (NH_4_)_2_SO_4_ | Timecourse (times vary for conditions); H_2_O_2_ adapted vs. Light-irradiated; Batch bioreactors | ~5.3 g/L, 33% untreated;  ~11.1 g/L 65% H_2_O_2_ treated | 3,786 | Catalase upregulated during nitrogen limitation. Modeling and proteomics show acetyl-CoA synthase and phosphoketolase ultimately supply acetyl-CoA in xylose condition. | Pinheiro et al. (2020) |  |
| Label-free Cellular Proteomics | - | Nitrogen | *R. toruloides* | 40 g/L Glucose 40 g/L Xylose | YNB (NH_4_)_2_SO_4_ | Timecourse (varying time points for glucose vs. xylose); Batch bioreactors | 6.56 g/L, 47.5% Glucose;  5.22 g/L, 47.1% Xylose | > 2,999^‡^ | Alcohol/aldehyde dehydrogenases is important for NADPH generation. Beta-oxidation enzymes were upregulated on xylose compared to glucose (futile lipid cycle). | Tiukova et al. (2019) |  |
| Label-free Cellular Proteomics | G,T | Nitrogen | *R. toruloides* | 70 g/L Glucose | YE (NH_4_)_2_SO_4_ | Timecourse (Seed†, 24 and 96h); Batch bioreactor (Technical replicates?) | 0.07 g/L,  22.8% High N; 0.16 g/L,  33.3% Low N | 3,108 | First systems-level construction of metabolism linked to oleaginous phenotype in this yeast. Found a new class of perilipin-like protein that likely protects lipid droplets. | Zhu et al. (2012) |  |
| Multiplexed Cellular Proteomics | M,T | Phosphate | *R. toruloides* | 27 g/L Glucose | (NH_4_)_2_SO_4_ | ~17 and ~60h for Pi replete and limited, respectively (steady-state); Chemostat | 0.02 g/L, 7.3%;  0.36 g/L, 43.9% | 4,212 | Pi limitation upregulates RNA degradation and downregulates PPP--NADPH regenerated by ME overexpression. | Wang et al. (2018) |  |
| Isotopic Dimethyl Labeling Phospho | - | Phosphate | *R. toruloides* | 27 g/L Glucose | (NH_4_)_2_SO_4_ | ~17 and ~60h for Pi replete and limited, respectively (steady-state); Chemostat | 0.02 g/L, 7.3%;  0.36 g/L, 43.9% | 5,659^PTM^ 3,556 | Phosphorylation of autophagy-related proteins observed; knockdown of ATG9 reduced lipid accumulation during Pi limitation. | Wang et al. (2023) |  |
| Label-free Lipid Droplet Proteomics | - | Phosphate Nitrogen | *R. toruloides* | 70 g/L Glucose | YE (NH_4_)_2_SO_4_ | 24h Nitrogen-rich, 24h Nitrogen-limited, 48h Phosphate-limited; Batch | 8.6% High N; ~20% Low N; 22.3% Low Pi | 226^§^ | Rab GTPases, coatomer components, perilipin-like protein (LDP1), and a caleosin family protein (vacuole interactions) were observed. | Zhu et al. (2015) |  |
| SILAC | G,T | - | *S. bombicola* | 120 g/L Glucose | YNB AA Mix | Timecourse (35h and 75h); Batch flasks | - | 615 | CYP450 catalyzes first step of sophorolipid synthesis, only observed in stationary phase. CYP regulator DAP1 upregulated. | Ciesielska et al. (2013) | |

| **Approach** | **Multi-omics** | **Stress** | **Organism** | **Carbon** | **Nitrogen** | **Experimental** | **Lipid Titer/Content** | **IDs** | **Significant Findings Summary** | **Ref.** |  |  |  |
| --- | --- | --- | --- | --- | --- | --- | --- | --- | --- | --- | --- | --- | --- |
| Label-free Secretomics | - | - | *S. bombicola* | 100 g/L Glucose | YE Urea | Timecourse (18, 30, 72 and 100h); Batch flasks | - | 44^§^ | Identified and characterized (knockout) lactone esterase involved in last step of sophorolipid synthesis. | Ciesielska et al. (2014) | |  |  |
| Label-free Cellular Proteomics | - | - | *X. dendrorhous* | 20 g/L Glucose | NH₄NO₃ | Timecourse (24, 70, and 96h); Batch flasks | - | 131^§^ | Developed a sample preparation approach to solubilize membrane proteins. Astaxanthin synthesis appears to play a primary role as an antioxidant defense mechanism. | Martinez-Moya et al. (2011) |  |  |  |
| Label-free Cellular Proteomics | M | - | *X. dendrorhous* | 20 g/L Glucose 20 g/L Succinate | NH₄NO₃ | Timecourse (24, 48, 70, and 96h); Batch flasks | - | 329^§^ | Upregulation of proteins like monooxygenase, cytochrome P450, phosphoglucomutase (trehalose production), and GAPDH aligns with carotenoid production. | Martinez-Moya et al. (2015) |  |  |  |
| Label-free Lipid Droplet Proteomics | - | - | *Y. lipolytica* | 20 g/L Glucose 5 g/L Oleic acid | YE Peptone YNB NH_4_Cl | Timecourse (3 and 24h); YPD vs. minimal medium with oleic acid; Batch flasks | - | > 30^‡,§^ | RAB GTPases involved in membrane trafficking, autophagy, etc. associated with LD. OIL1 protects LD from lipases. | Athenstaedt et al. (2006) | | |  |
| Label-free Cellular Proteomics | - | - | *Y. lipolytica* | Switchgrass hydrolysate | (NH_4_)_2_SO_4_ AA Mix | Timecourse (~8, ~12, ~46, and ~70h); Conventional vs. xylose-using strain; Batch bioreactor | ~2 g/L for both strains; No CDWs for content calc. | > 1,300^‡^ | The xylose-utilizing strain upregulates enzymes in PPP and differentially expresses lipid metabolic regulators (e.g., YAS2). | Walker et al. (2021) |  |  |  |
| Label-free Cellular Proteomics | - | - | *Y. lipolytica* | Catalytically depolymerized polyethylene | YNB (NH_4_)_2_SO_4_ | 24h or 48h; Oil-bound vs. floating cells; Batch flasks | 10.1%; No CDWs for lipid titer calc.; 2.33 g/L citric acid | > 1,000^‡^ | Some CYP450 and ALDH uniquely observed grown on hydrocarbons. Investment in lipid transport and metabolism at expense of growth. | Walker et al. (2023) |  |  |  |
| Label-free Cellular Proteomics | M | - | *Y. lipolytica* | 10 g/L Glucose  10 g/L Malate | YE Peptone | Multiple mutant strains compared; Batch flasks | Itaconic acid titers not provided for proteomics experiment | - | Bayesian metabolic modeling of integrated multi-omics data successfully identified enzymes for improving itaconate production. | McNaughton et al. (2021) | | | |
| Label-free Cellular Proteomics | - | - | *Y. lipolytica* | 40 g/L Glucose 5 g/L Phenylalanine | YNB (NH_4_)_2_SO_4_ | 24h; Phenylalanine supplemented vs. control; Batch flasks | 0.14 g/L  2-phenylethanol | 603 | Identified enzymes involved in 2-phenylethanol production, such as ARO10 responsible for phenylpyruvate decarboxylation, and others in Ehrlich pathway. | Celińska et al. (2015) |  |  |  |
| Selective (MALDI-TOF-MS/MS) | - | - | *Y. lipolytica* | 10 g/L Glucose | YNB GlcNAc | 24h; Yeast cells compared to hyphal cells (HOY1 null); Batch flasks | - | 600^§^ | Dimorphism studied using mutants and medium for hyphal growth induction. Differentially expressed proteins involved in carbohydrate and purine metabolism. | Morín et al. (2007) |  |  |  |

| **Approach** | **Multi-omics** | **Stress** | **Organism** | **Carbon** | **Nitrogen** | **Experimental** | **Lipid Titer/Content** | **IDs** | **Significant Findings Summary** | **Ref.** |  |  |  |  |
| --- | --- | --- | --- | --- | --- | --- | --- | --- | --- | --- | --- | --- | --- | --- |
| Label-free Cellular Proteomics | - | Nitrogen | *Y. lipolytica* | 40 g/L Glycerol | YE Peptone (NH_4_)_2_SO_4_ | Timecourse (9, 12, and 48h); Time-matched†; Batch flasks | - | > 1,000^‡^ | Minimal abundance changes to enzymes (e.g., ATP-citrate lyase) involved in routing carbon to fatty acid synthesis. | Sestric et al. (2021) | | | |  |
| Label-free Cellular Proteomics | M,T | Carbon Nitrogen | *Y. lipolytica* | Varies glucose | (NH_4_)_2_SO_4_ AA Mix | DGA1 overexpressing strain vs. reference genetically-modified strain; Chemostat | ~8% Ref. strain; ~20% DGA1 over-expressing strain | > 2,653^‡^ | DGA1 overexpression with nitrogen limitation modulates carbon flux to isopropyl-malate and leucine causing a complex interplay among TORC1, LEU3, autophagy. | Kerkhoven et al. (2017) | | | | |
| Label-free Acetylomics | - | Nitrogen | *Y. lipolytica* | 60 g/L Glucose | YE | 72h; Qualitative; Batch flasks | 4.5 g/L, 41.6% | 3,163^PTM^ 1,428 | Several enzymes involved in lipid metabolism (e.g., FAS) had complex acetylation patterns. | Wang et al. (2017) | |  |  |  |
| Multiplexed Cellular Proteomics | - | Nitrogen | *Y. lipolytica* | 25 g/L Glucose | (NH_4_)_2_SO_4_ | ~83 and ~50h (steady-state); High vs. low-lipid producing strains; Chemostat | 0.95 g/L, 27%;  no info. for 0.1  h^-1^ dilution rate or other strain | 4,441 | Separate biotin synthase and HOG1 deletions increased lipid content by ~20%. | Poorinm-ohammad et al. (2022) |  |  |  |  |
| Multiplexed Phospho | M | Nitrogen | *Y. lipolytica* | 25 g/L Glucose | YNB (NH_4_)_2_SO_4_ | 9h; Nitrogen rich vs. limited comparison; Batch flasks | - | 1,219^PTM^ 4,926 | Many phosphorylation sites of kinases not conserved in *S. cerevisiae*. RIM11 phosphorylation conserved and regulates glycogen synthesis. | Pomraning et al. (2016) | | |  |  |
| Label-free Cellular Proteomics | - | Thiamine | *Y. lipolytica* | 20 g/L Glucose | (NH_4_)_2_SO_4_ | Timecourse (~10 and ~16h^‡^); Batch flasks | - | > 1,600^‡^ | Engineered de novo thiamine synthesis pathway. Deficiency affects enzymes in ETC and lipid metabolism. | Walker et al. (2020) |  |  |  |  |

Details are only provided for the actual samples that were processed for proteome profiling. In the "Multi-omics?" column, the letters correspond to the following: genomics (G), transcriptomics (T), metabolomics (M), and lipidomics (L). Refer to the manuscripts for carbon and nitrogen sources that were separately compared (in some cases a mixture of carbon sources like glucose and xylose was studied). In many cases, there isn't corresponding lipid metrics. For filamentous fungi, PUFA lipid results are provided when specified. Note that the number of unique proteomics IDs is heavily influenced by sample processing, incorporation of offline fractionation to reduce sample complexity, and the MS searching algorithm (i.e., using MaxQuant with match-between-runs boosts IDs but many of them have missing values). Refer to the main text for reference citations.

^†^ Seed culture medium (YPD) or medium for comparison differs substantially from the other media used in the experimentation

^‡^ Exact number not reported but estimated from differential expression results or inferred from supplemenatry material

^§^ SDS-PAGE gel bands

^PTM^ Number of unique PTM sites (the number of protein IDs is provided below)

~ Exact number not reported but extracted from figure(s)
